# Identification of a direct interaction between the Fab domains of IgG antibodies and human FcRn upon IgG-FcRn complex formation

**DOI:** 10.1038/s42003-025-08252-z

**Published:** 2025-06-12

**Authors:** Esben Trabjerg, Jeannette Nilsen, Torleif Tollefsrud Gjølberg, Jan Terje Andersen, Alexander Leitner, Kasper D. Rand

**Affiliations:** 1 Department of Biology, Institute of Molecular Systems Biology, ETH Zürich, Zurich, Switzerland; 2 Precision Immunotherapy Alliance (PRIMA), University of Oslo, Oslo, Norway; 3 Department of Immunology, Oslo University Hospital Rikshospitalet and University of Oslo, Oslo, Norway; 4 Institute of Clinical Medicine, Department of Pharmacology, University of Oslo and Oslo University Hospital, Oslo, Norway; 5 Department of Pharmacy, University of Copenhagen, Copenhagen, Denmark; 6 Present Address: Department of Pharmacy, University of Copenhagen, Copenhagen, Denmark

**Keywords:** Structural biology, Protein design

## Abstract

IgGs have become successful drug scaffolds by combining specific target binding with the ability to induce cellular cytotoxicity. Furthermore, IgGs possess unusually long half-lives in the blood (2-3 weeks). IgGs achieve such extraordinary half-lives through a pH-dependent interaction with the FcRn-receptor whereby IgGs are recycled. No high-resolution structure of FcRn in complex with a full-length IgG is available, and the interaction was long thought to be mediated solely via the IgG-Fc. However, some IgGs with identical Fc-parts, but different Fab-domains, exhibit different half-lives, suggesting involvement of the Fab-domains in FcRn binding. Here, we employ structural mass spectrometry (HDX-MS and XL-MS) to explore the interaction of full-length IgGs with FcRn. HDX-MS and XL-MS experiments confirm an interaction between FcRn and the Fc-region of IgGs, through three cross-links between FcRn and the IgG-Fc-domain and a reduction in HDX in both the receptor and the Fc-region upon complex formation. However, FcRn-induced changes in HDX are also observed in the Fab-domains, supported by cross-links between the Fab-domains and the α3-domain of FcRn. Our results thus provide direct evidence for an IgG Fab-FcRn interaction. We envision that these results could advance the engineering of IgG-antibodies with tailored pharmacokinetics and enhanced efficacy.

## Abstract (web summary)

Two orthogonal mass spectrometry techniques reveal that both the Fc and Fab domains of IgGs make direct interactions with FcRn, which could inform the engineering of new improved therapeutic IgGs.

## Introduction

Since the first monoclonal antibody (mAb) was approved for clinical use in 1986^1,2^, mAbs have evolved to become the fastest growing class of protein-based therapeutics. The reason for this is an improved biological understanding that has been combined with advances in discovery, engineering, and manufacturability. This has resulted in more than 100 approved mAbs by the FDA and impressively more than 1200 candidates under evaluation^3,4^. While the pipeline is still dominated by mAbs targeting cancers, followed by chronic inflammatory diseases, modalities have also been developed to treat conditions and diseases such as transplantation rejection, infectious diseases, cardiovascular diseases, hemophilia, and even severe migraine.

The vast majority of therapeutic mAbs are of the Immunoglobulin G (IgG) isotype, and in particular the IgG1 subclass. The IgG1 subclass combines highly specific antigen binding with the ability to mediate effector functions such as antibody-dependent cytotoxicity, complement-dependent cellular cytotoxicity, and antibody-dependent cellular phagocytosis, or alternatively engineered to have reduced or abolished capacity to induce these functions^5–7^.

A further advantageous characteristic is their unusually long half-life in humans of three weeks on average, whereas similarly sized soluble proteins typically have half-lives of days^8,9^. The extraordinarily long half-life is due to a pH-dependent interaction with the neonatal Fc receptor (FcRn), which is broadly expressed in both hematopoietic and non-hematopoietic cells^10–18^. FcRn binds IgG at slightly acidic conditions (pH 5.0–6.5), such as in early endosomes following fluid-phase pinocytosis, while the interaction is negligible at the neutral pH (pH 7.4) of the extracellular space. For instance, in endothelial cells lining the vascular system, FcRn rescues endocytosed IgGs from lysosomal degradation by binding to antibodies in the acidic environment of early sorting endosomes and redirecting them to the cell surface^19,20^. Here, the neutral pH of the circulation abolishes the interaction between the receptor and IgGs, resulting in release of the IgGs back into the bloodstream^21,22^.

FcRn is a transmembrane heterodimer with a similar structural architecture to major histocompatibility complex (MHC) I proteins, but instead of presenting antigenic peptides to cytotoxic T cells, FcRn has evolved to engage IgG and human serum albumin (HSA) via separate binding sites^11,23–25^. Specifically, FcRn consists of a heavy chain (HC) with three extracellular domains, namely α1, α2, and α3, followed by a transmembrane helix and a C-terminal cytoplasmic tail. The extracellular domains of the HC are non-covalently associated with the light chain (LC), β-2 microglobulin (β2m)^23,26–28^. The α1 and α2 domains form a fold of two α-helices on top of a seven-stranded β-sheet, while the membrane proximal α3 domain and β2m share structural homology with immunoglobulin domains^23,26–28^.

IgGs consist of two heavy chains (HCs) and two light chains (LCs) joined together by interchain disulfide bonds, forming a “Y-shaped” protein^29,30^. The HC consists of a variable region (VH) and three constant regions (CH1, CH2, and CH3), while the LC consists of a variable region (VL) and a single constant region (CL). The arms of the “Y-shaped” structure are referred to as the antigen-binding fragment (Fab; VH, CH1, VL, and CL), while the stem of the “Y-shaped” structure is referred to as the fragment crystallizable region (Fc; CH2, and CH3). The Fab domains are responsible for recognizing and binding to antigens, while the Fc region is responsible for immunogenic effector functions^29,30^.

Based on interaction studies and solved crystal structures of truncated FcRn in complex with IgG Fc fragments, the principal binding site for FcRn has been mapped to the elbow region between the CH2 and CH3 domains, where one FcRn molecule can bind to each side of the Fc^10,25,28,31^. However, we and others have demonstrated that biophysical properties, such as charge patches and isoelectric point, as well as introduction of amino acid substitutions in the distal Fab domains, can modulate receptor binding and dramatically affect the half-lives of IgG1s and IgG1 Fc containing molecules in mice, non-human primates, and humans^9,32–36^. This may explain why the plasma half-life of therapeutic IgG1 molecules varies greatly in humans, from strikingly 6 to 32 days^1^. One example is ustekinumab and briakinumab, both IgG1 molecules binding the same antigen and having identical amino acid composition in the canonical binding site for FcRn, which exhibit markedly different half-lives of 22 days and 8–9 days, respectively^34^. This is a direct result of differences in charge patches of their variable regions, CDRs, where briakinumab is more positively charged, which greatly affects cellular uptake and FcRn-mediated transport^36^. Thus, while the Fc contains the principal FcRn binding site, these data support that the Fab domains can also modulate receptor binding and transport behavior.

While there is no solved high-resolution structure of FcRn in complex with a full-length IgG available, FcRn chromatography, molecular dynamics simulations, hydrogen/deuterium exchange mass spectrometry (HDX-MS), negative stain electron microscopy and hydroxyl radical footprinting MS have revealed a possible direct interaction between the Fab domains and the membrane proximal α3 domain of FcRn^33,34,37,38^. However, direct experimental proof of such a molecular interaction has so far remained elusive. Thus, there is a need to gain a better in-depth understanding of how differences in the variable Fab domains contribute to and affect FcRn engagement. This is not only of importance from a biological point of view, but also regarding the design and selection of lead mAb candidates with favorable pharmacokinetic properties.

In this study, we delineated the binding mode between a soluble truncated version of human FcRn and two distinct full-length IgG1 molecules by a combined structural MS approach, specifically cross-linking MS (XL-MS) and HDX-MS. Our results support that when FcRn engages the Fc of a full-length IgG1 at acidic pH, the Fab domains are positioned at a suitable distance and with an orientation allowing proximity with the membrane proximal α3 domain of FcRn, where direct interaction may occur via both the LC and HC of the IgG. Our findings provide new molecular insights into how FcRn engages full-length IgG molecules, which should inform optimized engineering of IgGs with tailored pharmacokinetics.

## Results

### Sequence analysis of two IgG mAbs

To study the influence of Fab domains on binding to FcRn, we chose to investigate the complexes formed between a truncated form of human FcRn and two different mAbs; a fully human IgG1 (Adalimumab; H-mAb) with specificity for tumor necrosis factor alpha^39^, and a mouse-human chimeric IgG1 with specificity for the hapten 4-hydroxy-3-nitrophenylacetate (C-mAb)^40^. H-mAb contains a human HC and a human κ LC, while C-mAb contains a human HC and a mouse λ LC^40,41^. To investigate the differences between the mAbs, their amino acid sequences were aligned^42–44^. The alignment showed that the two mAbs share identical HC constant domains, while having distinct differences in the HC Fab domains (Supplementary Figs. S1 and S2). Specifically, H-mAb contains a residue more in the CDR3 region compared to C-mAb. Hence, to ease the comparison of the obtained XL-MS and HDX-MS results from the two different complexes, we aligned the two sequences and chose to skip residue number 104 in the HC of C-mAb in accordance with the alignment (Supplementary Fig. S1). The sequence alignment also revealed that the LCs are 41% identical, while the variable domains of the HCs are 50% identical (Supplementary Fig. S2)^42–44^.

Differences in surface charge have earlier been shown to affect the half-life and binding affinities of IgGs toward FcRn^34,36^. Hence, we performed a comparison of the net charge of the Fab domains of the two mAbs at pH values spanning the pH in endosomes and the blood (Supplementary Fig. S3). No clear difference in the net charge of the Fab domains of H-mAb or C-mAb was detected. Furthermore, we also analyzed the surface potential of the two Fab domains at pH 6.0 (pH of endosomes) and at pH 7.4 (pH of the blood stream) (Supplementary Fig. S3). Here, only minor differences in the surface potential were observed. Finally, we have earlier shown that the two mAbs have similar binding affinity toward FcRn at pH 5.5, and that they could not be distinguished by FcRn-chromatography^35^.

### XL-MS demonstrates a direct interaction between the Fab domains of both mAbs and FcRn

To investigate the putative interaction between the Fab domains of IgGs and the C-terminal α3 domain of the FcRn molecule upon FcRn:IgG complex formation, we performed a structural analysis of the complex by XL-MS. In XL-MS, the protein complex is treated under native conditions with a chemical reagent that cross-links amino acid side chains. After cross-linking and enzymatic digestion, cross-linked peptides can be identified by liquid chromatography coupled with high-resolution MS. The identity of cross-linked residues and the length of the cross-linker can be used to provide “molecular rulers” that translate into atomic distance restraints, which can be used for integrative modeling^45–49^.

In the current study, disuccinimidyl suberate (DSS) was used as the primary cross-linking agent, which is an amine reactive cross-linker where the cross-linking reaction is typically performed at pH 7.5 or above to secure sufficient cross-linking yield^50,51^. However, we have very recently shown the ability of DSS to cross-link lysine residues even at slightly acidic conditions, paving the way for a cross-linking analysis of FcRn in complex with full-length IgGs at pH 6.0^52^.

**Fig. 1:**
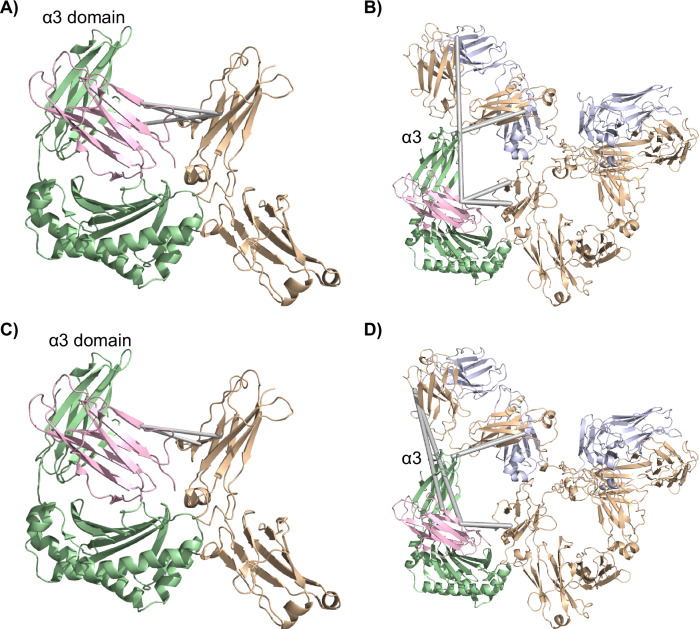
Identification of inter-protein cross-links between FcRn and IgGs. **A** and **C** Identified cross-links between FcRn and the Fc-part of H-mAb (**A**) or C-mAb (**C**) are visualized on the crystal structure of FcRn in complex with Fc-YTE (PDB ID: 4N0U. HSA was omitted to simplify the figure)^25^. **B** and **D** Identified cross-links between FcRn and H-mAb or C-mAb are visualized onto homology models of C-mAb and H-mAb aligned with Fc-YTE in PDB structure 4N0U. *Pale green*: HC of FcRn, *light pink*: β2m, *wheat*: Fc-YTE and HC of C-mAb and H-mAb, *light blue*: LC of H-mAb and C-mAb, *gray* lines: identified inter-protein cross-links. *α3* denotes the α3 domain on (**B**) and (**D**).

To secure full sequence coverage, especially of the α3 domain of FcRn, two different digestion protocols were explored: one with a combination of LysC and trypsin and one with AspN^53^. With this experimental setup, six inter-protein cross-links in the FcRn:H-mAb complex were identified (Table 1 and Supplementary Table S1). Three of the cross-links involving K6 and K91 of the β2m subunit of FcRn and K292 and K294 of the CH3 domain of the H-mAb Fc were identified (Fig. 1A). This is in accordance with published X-ray crystal structures of truncated forms of IgGs in complex with FcRn, where the canonical interaction site is found between the CH2-CH3 elbow region of the Fc and the α2 domain of FcRn (Fig. 1A)^25,28^. Furthermore, two cross-links were identified between the Fab domains and the α3 domain of FcRn, specifically between K217 and K218 of the IgG HC and K243 of the receptor (Fig.1B). In addition, one cross-link between the N-terminal amino group of the IgG and K91 of the β2m subunit of FcRn was detected (Fig. 1B).

**Table 1:** Identified inter-protein cross-links between FcRn and H-mAb or C-mAb

	Residue 1	Residue 2	ld-score	FcRn: H-mAb	FcRn: C-mAb	Euclidean Cα-Cα distance (Å)
1	β2m - K91	Fc HC - K292	34.11	2/2	5/5	16.8
2	β2m - K91	Fc HC - K294	36.36	1/2	4/5	20.5
3	β2m - K6	Fc HC - K292	33.26	2/2		24.0
4	α3 - K243	Fab HC - K217	36.16	1/2	3/3	
5	α3 - K243	Fab HC - K218	40.56	2/2	3/3	
6	β2m - K91	Fab HC – K59	33.41		2/5	
7	β2m - K94	Fab HC – K59	37.86		4/5	
8	β2m - K91	Fab HC – E1 (N-term)	32.60	2/2		

The highest ld-score observed across all data sets is shown. The numbers in the FcRn:H-mAb and FcRn:C-mAb columns refer to how many times the cross-links were observed in experimental replicates. Euclidean Cα-Cα distances (Å) refer to the solved crystal structure of FcRn in complex with the YTE containing Fc fragment (PDB ID: 4N0U). α3 refers to domain α3 of the FcRn HC.

Moreover, when the same XL-MS experiment was performed with the FcRn:C-mAb complex, a total of six cross-links were identified (Table 1). Two of these connected K292 or K294 of the IgG Fc and K91 of the β2m subunit of FcRn (Fig. 1C), as observed for the FcRn:H-mAb complex (Fig. 1A). The other four cross-links connected the Fab domains of the C-mAb with the receptor, including the two identified in the FcRn-H-mAb complex, which was between K217 and K218 of the IgG HC and K243 of the α3 domain of FcRn (Fig. 1D). The additional two cross-links were detected between K59 of the C-mAb Fab domains and K91 and K94 of the β2m subunit of FcRn (Fig. 1D). Thus, two of the cross-links connecting the Fc-part with β2m, as well as two of the cross-links connecting the Fab domains of the IgGs with the α3 domain of FcRn, were identical between the two investigated FcRn-IgG complexes. The data support that H-mAb and C-mAb bind FcRn in a similar orientation, involving both the Fc and the Fab domains of the two IgG1s, despite considerable sequence differences in the Fab domains of the two mAbs (human κ LC vs. mouse λ LC, Supplementary Fig. S2).

### Visualization of identified cross-links

The identified cross-links between FcRn and the IgG Fc were visualized onto the crystal structure of human FcRn in complex with human Fc-YTE (4N0U) (Fig. 1A, C)^25^. The Euclidean C_α_-C_α_ distances between the cross-linked residue pairs are all below a distance cut-off for DSS cross-linking of 30 Å, used in the current study^54,55^.

No high-resolution structure has to our knowledge been published of FcRn in complex with a full-length IgG. Thus, to build a homology model, we explored high-resolution structures of six different full-length IgGs that have been published (PDB ID: 1MCO, 1IGT, 1IGY, 1HZH, 5DK3, 6GFE)^56–61^. The single domains of the structures are highly similar, but the orientation of the Fab domains relative to the Fc-part is quite different across the six structures, indicating considerable flexibility in the hinge region connecting the Fab domains to the Fc-part (Supplementary Fig. S4). By overlaying the six available crystal structures of a full-length IgG with the Fc-YTE bound to FcRn in the solved co-crystal structure, we found that the orientation of the Fab domains in the “Y-shaped” IgG conformation of 1IGY aligned the best with the XL-MS data, while avoiding steric clashes between the α3 domain of FcRn and the Fab domains (Supplementary Fig. S4). Hence, the identified cross-links were visualized onto a homology model of H-mAb and C-mAb that was built with the “Y-shaped” conformation of 1IGY as a template (see “Methods” section, Fig. 1B, D). However, multiple of the cross-links exceeded the 30 Å threshold value for DSS cross-links, indicating that further rotations and movement of the Fab domains are needed to bring these closer to the α3 domain and β2m of FcRn and for the model to fit the experimental XL-MS data. In support of this notion, we observed several intra-protein cross-links that connected the Fab domains to the Fc-part of both H-mAb and C-mAb, which are not compatible with a rigid IgG “Y-shape” (Supplementary Table S2).

### Cross-linking of FcRn:IgG complexes with alternative cross-linking chemistries

To increase the amount of structural information, we also cross-linked the FcRn:C-mAb complex with two different cross-linking chemistries targeting acidic residues^62,63^. Several intra-protein cross-links were identified, highlighting the ability of both chemistries targeting acidic residues to cross-link proteins at slightly acidic conditions. However, no credible inter-protein cross-links were identified (Supplementary Table S3 and Supplementary Fig. S5).

Furthermore, we studied a Fc-engineered version of C-mAb (mutC-mAb) containing five amino acid substitutions (M255Y, S257T, T259E, H436K, and N437F) that binds more strongly and less pH dependently to FcRn than C-mAb^40,64,65^. The mutC-mAb:FcRn complex was cross-linked by DSS and the two chemistries targeting acidic residues described above. The results revealed cross-links similar to those already identified between FcRn and H-mAb or C-mAb, but also the appearance of a multitude of additional cross-links between the Fab domains and all four domains of FcRn (α1, α2, α3, and β2m) (Supplementary Table S3 and Supplementary Fig. S5). However, we were unable to identify experimental conditions, in which we did not observe the appearance of higher-order oligomer states during the cross-linking reactions. Hence, we dismissed these cross-links in the further structural analysis of FcRn in complex with H-mAb or C-mAb (Supplementary Fig. S6).

### Control experiments confirm the specificity of the FcRn-IgG interaction

To further confirm that the identified DSS cross-links were not due to unspecific interactions and reflected the solution-phase structure of the FcRn:IgG complex, we performed two different control experiments. First, we treated C-mAb with IdeZ, an enzyme that cleaves IgGs at the hinge region, generating two Fab fragments and one Fc-part^66^. IdeZ-treated C-mAb or IdeZ-treated mutC-mAb were mixed with FcRn, and the resulting mixture was cross-linked with DSS and subsequently digested with AspN and Trypsin, respectively. No cross-links between the Fab domains of either C-mAb and mutC-mAb and FcRn could be detected, while cross-links between the Fc-part of mutC-mAb and FcRn could be detected (Supplementary Table S4). Secondly, we cross-linked a mixture of free β2m and C-mAb, as β2m has been observed to bind unexpected binding partners^67^. Again, no cross-links were identified between C-mAb and β2m in the absence of the rest of the receptor. Thus, the control experiments support that the FcRn-Fc interaction is a prerequisite to allow detectable Fab domain interaction with FcRn. In addition, the identified interaction between the Fab domains and FcRn is not due to the unspecific binding of β2m to the Fab domains.

### Interaction space analysis reveals that the Fab domains are in close proximity to the α3 domain and β2m of FcRn

To visualize the dynamic position of the Fab domains in accordance with the XL-MS constraint, we performed an interaction space analysis with the software tool DisVis^68,69^. The results of the analysis of cross-linking constraint from the H-mAb:FcRn complex revealed a single interaction space consistent with a single conformation (Supplementary Fig. S7). The resulting accessible interaction space showed a sphere hovering above the α3 domain and β2m of the FcRn molecule. Similar results were obtained for the C-mAb cross-links. Importantly, the distance constraints from C-mAb or H-mAb in complex with FcRn generate an overlapping interaction space (Supplementary Fig. S7).

### HDX-MS shows changes in the conformational dynamics of the Fab domains upon FcRn complex formation

While XL-MS reveals the spatial proximity of amino acid side chains, HDX-MS probes the conformational dynamics of the entire protein backbone, except at proline residues, by measuring the HDX rate of backbone amides when exposed to deuterated solvent^70–74^. HDX rates of amide hydrogens in a protein are determined by hydrogen bonding status and strength, and to a lesser extent, solvent accessibility^75,76^. Fully solvated (non-hydrogen bonded) amides exchange rapidly, while amides in structured regions (hydrogen bonded) can exchange up to eight orders of magnitude slower^77^. Protein interactions and their conformational impact can be mapped by HDX-MS as binding perturbs HDX rates, due to changes in hydrogen bonding status and solvation directly in the binding interface or indirectly in conformationally linked regions^74,78^.

**Fig. 2:**
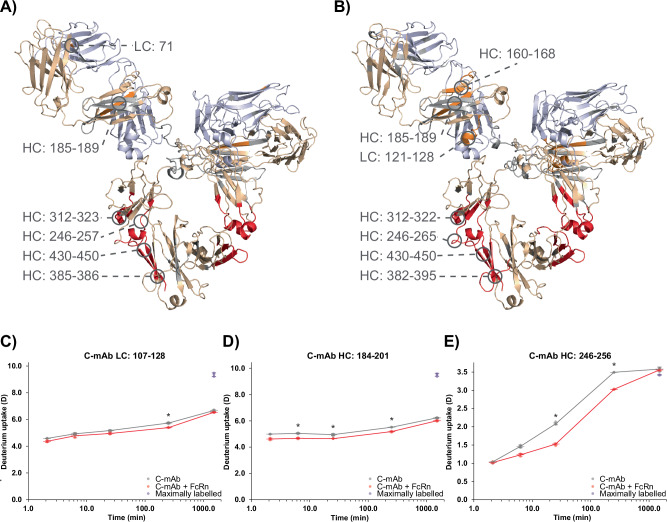
Conformational response of H-mAb and C-mAb to FcRn binding. **A** and **B** Differential HDX-MS results mapped onto homology models of H-mAb (**A**) and C-mAb (**B**). Regions in the Fab domains and the Fc-part of the mAbs displaying a significant protection from exchange in the presence of FcRn are colored *orange* and *red*, respectively. LCs are colored *light blue*, HCs are colored *wheat*, and regions for which no HDX information could be obtained are colored *gray*. **C**–**E** HDX plots of selected peptides. HDX is plotted as a function of time for C-mAb (*gray* curves) and C-mAb in the presence of FcRn (*red* curves). Maximally labeled (90%) FcRn samples are plotted as *purple* spheres at the longest time point. Single values are shown as spheres, the mean is shown as a long horizontal line, and the SD is plotted as error bars. An asterisk marks time points with a significant difference in HDX. (*n* = 3).

To gain a better understanding of the conformational dynamics of IgG-FcRn complex formation, we analyzed the FcRn:H-mAb and FcRn:C-mAb complexes by HDX-MS. First, we explored the binding impact of FcRn on C-mAb or H-mAb. HDX data could be retrieved for a total of 100 peptides for C-mAb and 128 peptides for H-mAb, resulting in a sequence coverage above 88% for both mAbs (Supplementary Figs. S8 and S9). The most pronounced changes in HDX upon FcRn binding to the mAbs were in the canonical FcRn binding region in the CH2-CH3 elbow region of the Fc-part, with only minor differences between the mAbs (Fig. 2). Specifically, a significant decrease in HDX was observed in regions corresponding to: residue 246–265, 312–323, 382–395, and 430–450. However, the changes in HDX were not confined to the Fc, as significant changes could also be observed in the Fab domains. While two separate regions in the H-mAb Fab domains showed a significant decrease in HDX (residue 71 of the LC and residues 185–189 of the HC, Fig. 2A), three regions in the Fab domains of C-mAb showed a significant decrease in HDX (residues 121–128 of the LC and residues 159–167 and 184–188 of the HC, Fig. 2B). A slight protection from HDX was observed in peptides spanning residues 149-178 of the HC of H-mAb and in peptides spanning residues 50-68 of the LC of C-mAb. These effects were, however, not significant according to the threshold set for this study. Thus, the only clear difference between the conformational response of H-mAb and C-mAb to FcRn binding was the protection from exchange in residues 121–128 seen in the LC of C-mAb (Fig. 2. and Supplementary Figs. S10 and S11).

**Fig. 3:**
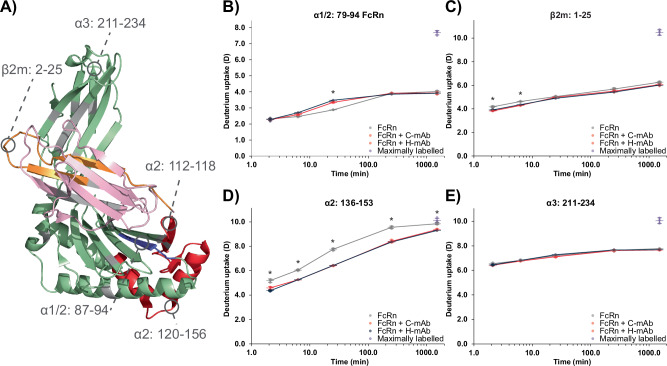
Conformational response of FcRn to H-mAb or C-mAb binding. **A** Differential HDX results mapped onto the crystal structure of FcRn (α-domain: *pale green*, β2m: *light pink*, PDB ID: 4N0U). Regions in the α domain and β2m displaying a significant protection from exchange in the presence of H-mAb or C-mAb are colored *orange* and *red*, respectively. Regions displaying a significant deprotection from exchange in the presence of H-mAb or C-mAb are colored *blue*, while regions for which no HDX information could be obtained are colored *gray*. **B**–**E** HDX uptake plots of selected peptides. HDX is plotted as a function of time for FcRn (*gray* curves), FcRn in the presence of C-mAb (*red* curves), and FcRn in the presence of H-mAb (*blue* curves). Maximally labeled (90%) FcRn samples are plotted as *purple* spheres at the longest time point. Single values are shown as spheres, the mean is shown as a long horizontal line, and the SD is plotted as error bars. An asterisk marks time points with a significant difference in HDX (*n* = 3 for all data points, except the last four time points of FcRn in the presence of H-mAb, where *n* = 1).

Next, we explored the binding impact of H-mAb or C-mAb on FcRn, with exchange times ranging from 2.1 to 1507.13 min. We obtained HDX data from 45 peptides, resulting in a sequence coverage of the FcRn HC and β2m above 96% for both chains (Supplementary Fig. S12). Strikingly, the binding impact for both investigated mAbs was highly similar. A significant protection from HDX was observed in peptides spanning the canonical Fc binding region in the α2 domain, e.g., residues 112–118 and 121–156 (Fig. 3 and Supplementary Fig. S13). The binding impact was not confined to the FcRn HC, as a significant decrease in HDX was also observed in the N-terminal part of β2m (residue 2–25) (Fig. 3C).

Interestingly, two peptides spanning region 87–94 of the FcRn HC showed a significant increase in HDX upon binding both C-mAb and H-mAb, hinting at a slight rearrangement of the beta-sheet structure in the last residue of the α1 domain and the very first part of the α2 domain of the FcRn HC (Fig. 3B and Supplementary Fig. S14).

In stark contrast to the XL-MS data and HDX effects observed in the Fab domains of the IgGs, no significant differences in HDX could be observed in the α3 domain of FcRn. To further confirm this finding, we expanded the time window of the HDX-MS experiment to also include time points as early as 13 s, as several of the peptides spanning the top of FcRn (α3 domain) showed high incorporation of deuterium already at the earliest time point (Supplementary Fig. S13). Note that the HDX reaction was performed at pD_read_ = 6.0, hence, 2.09 min corresponds to approximately 5 s at pD_read_ = 7.4, due to the pH dependence of the chemical exchange rate (*k*_*ch*_). The shortest time point of 13 s corresponds to approximately 0.5 s at pD_read_ = 7.4^79–81^. Even at the shorter exchange times, no additional significant changes in HDX were observed in the α3 domain or in other regions of FcRn (Supplementary Fig. S15).

## Discussion

Here, we have performed a structural characterization of the complex between full-length IgG1s (H-mAb and C-mAb) and FcRn by a combination of XL-MS and HDX-MS. Our results from both XL-MS and HDX-MS confirm the presence of the canonical binding site between FcRn and the elbow region of the Fc, in good accordance with the published crystal structure of FcRn in complex with Fc-YTE, former HDX-MS analysis, and hydroxyl radical footprinting experiments of the complex between full-length IgGs and FcRn^33,37,38,82^. All identified cross-links between the IgG1 Fc and FcRn were below 30 Å (Euclidean Cα-Cα distances), based on the solved FcRn-Fc-YTE co-crystal structure. Furthermore, most regions with significant changes in HDX contain residues which belong to a large hydrophobic patch in the binding interface (FcRn residues Tyr88, Leu112, Phe117, Trp131, Pro132 and Leu135 and Fc-YTE residues corresponding to residue Leu251, Tyr252, Ile253, Leu309, and Leu314 of the HCs of the investigated mAbs) or directly engage in inter-molecular hydrogen bonds between the two binding partners (FcRn residues Glu115, Glu116, Gly129, Glu133, and Fc-YTE residues corresponding to residue Ile253, Thr254, His310, Gln311, and Asn434 of the HCs of H-mAbs and C-mAbs)^25^.

A structural stabilization is observed in a single region (residues 382–395) in the Fc-part of the mAbs upon FcRn binding, which cannot be directly rationalized based on the crystal structure of FcRn in complex with Fc-YTE. This region encompasses β-strand C and the C–D loop of the CH_3_ domain of H-mAb and C-mAb, and thus the conformational dynamics of this region could be closely linked to the conformational dynamics of β-strands F and G, which are stabilized in the presence of FcRn. Furthermore, the region is in close proximity to the actual binding interface, and similar conformational stabilization has been reported in three other studies investigating the conformational impact of FcRn binding on IgGs^33,38,82^.

While residue Y88 of FcRn is described as part of the hydrophobic patch between FcRn and Fc-YTE, no significant change in HDX has been observed in peptides covering this residue in former HDX studies^38^. In the current study, we observed an increase in HDX in peptides spanning this residue upon complex formation, pointing toward a higher flexibility of this region upon complex formation. To observe an increase in HDX and thereby an increase in conformational dynamics of a protein region upon complex formation is not common, however, it is not unheard of^83^. Interestingly, we have recently shown that flexibility in specific regions of domain III of HSA, another endogenous binding partner of FcRn, is important for efficient binding to the receptor^84^. More research is needed into this specific feature of the binding of FcRn to its two ligands to determine the importance of the observed phenomenon.

The identification of cross-links between the Fab domains and the α3 domain of FcRn is, to our knowledge, the first time an interaction between the Fab domains of an IgG1 and the α3 domain of FcRn has been confirmed experimentally in a direct manner (Fig. 1). However, it is important to note that a cross-link only confirms that the cross-linked residues have been in close proximity (<30 Å), not that the two residues directly interact.

In support of the identified cross-link between the Fab domains and the α3 domain of FcRn, a structural stabilization upon complex formation is not only observed in the Fc-part of the mAbs but also in the Fab domains (Fig. 2). Earlier studies have also identified the ability of the variable regions in the Fab domains of IgGs to affect FcRn binding, and two HDX-MS studies have described a structural stabilization in the Fab domains upon complex formation with FcRn^33,34,38,85,86^. Furthermore, a recent study used hydroxyl radical footprinting mass spectrometry to detect a decrease in solvent accessible surface area in both the Fab domains and the α3 domain of FcRn upon complex formation^37^.

However, in the current study, no structural stabilization was observed in the α3 domain of the FcRn molecule by HDX-MS. This can either be due to undersampling of the exchange time window or the fact that the interaction is transient with relatively low affinity, and/or from the FcRn point of view, is mainly driven by side chain interactions. The latter has earlier been suggested in the literature as the α3 domain of FcRn is highly negative, and that a concentration of positive charge on the Fab domains can cause excessive binding of IgG1s to FcRn at neutral pH^34^.

This observation further supports the combination of orthogonal structural methods in characterization of transient protein interactions, e.g., the fruitful combination of HDX-MS with XL-MS, where HDX-MS probes the conformational dynamics of the protein backbone while XL-MS reports on the proximity of side chains.

In the current study, we investigated the complex formation between two different IgG1s and FcRn. H-mAb and C-mAb only differ in their Fab domains (Supplementary Figs. S1 and S2), but despite these sequence differences, a highly similar binding impact on both FcRn and the respective IgG1s was observed by both XL-MS and HDX-MS upon complex formation (Figs. 2 and 3 and Supplementary Figs. S10, S11, S13, and S14). The presented data show that the implication of the Fab domains in the IgG1 interaction with FcRn is not only observed for human IgG1s but also present in a chimeric IgG1 construct. The observed effects in the Fab domains of the chimeric construct point toward that the interaction between the IgG1 Fab domains and FcRn is not only present in humans, but also found in other species. A feature that is important for correlating pharmacokinetic and -dynamic parameters of IgG1-based therapeutics measured in animal models to those in humans.

The structure of IgG1s is often depicted as a static “Y-shaped” structure. However, even early crystal structures of full-length IgG1s identified at least two separate conformations, namely the “Y-shape” and the “T-shape” (Supplementary Fig. S4)^87^. Furthermore, several other experimental methods, including in-solution methods, in combination with computational modeling, have highlighted that the Fab domains of IgG1s are highly flexible and can traverse a large conformational space relative to the Fc region^88–93^. The observed direct interaction between the α3 domain of FcRn and the Fab domains requires a high degree of flexibility of the hinge regions of the IgG1s. In agreement with this, it has been shown that the reason for decreased FcRn-mediated transcytosis of IgG2s is due to a missing glycine in its lower hinge, reducing flexibility of the hinge region^94^. The proposed direct interaction between the α3 domain of FcRn and the Fab domains of IgG1s might decrease the flexibility in the hinge region and result in decreased conformational dynamics. Unfortunately, we did not obtain sequence coverage of the hinge region in the HDX-MS experiments of the current study (Supplementary Figs. S8 and S9). Future HDX-MS studies with the use of complementary acidic proteases, such as Aspergillus niger Prolyl Endoprotease^95^ or nepenthensin^96–98^, possibly combined with electrochemical reduction of the cysteine bonds tying the hinge region together, could investigate this phenomenon further^99–101^. We consider the presence of an inter-domain allosteric signal between Fab- and Fc regions upon FcRn binding possible, but unlikely, due to the fact that the two impacted regions in Fab- and Fc regions are far apart and reside in different domains that have been shown to be able to move independently to each other – and we observe no HDX change in regions between the Fab- and Fc region. However, we note that it has been shown that antigen binding to an IgG1 can cause allosteric effects in the part of the Fc responsible for binding the Fcγ receptor^102^.

It is important to note that the current study was performed in vitro on a soluble version of FcRn. In its native environment, FcRn resides in the lipid membrane of the endosome. Here, the soluble extracellular part of FcRn is tethered to a transmembrane-spanning helix by a 10-residue linker. The binding stoichiometry and orientations between FcRn and IgGs are still debated in the literature^25,35,87,94,103^, but recent data from x-ray crystallography and negative stain electron microscopy have shown that a single IgG can engage two FcRn molecules in vitro^25,37^. This feature is also important in vivo as a decrease in half-life in rat and mouse models has been reported for IgG constructs, where only one half of the Fc-domain was able to bind FcRn^10^.

**Fig. 4:**
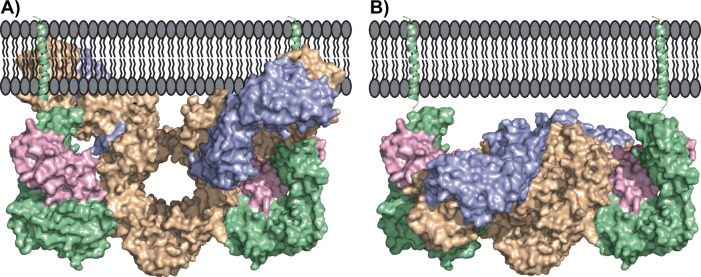
Structural model of IgG binding to FcRn in the context of the endosome membrane. **A** Model of a “Y-shaped” IgG1 binding to FcRn showing a clear steric clash between the Fab domains and the endosome membrane. **B** Model of a “T-shaped” IgG1 binding to FcRn showing no clash between the Fab domains and the endosome membrane. The models were produced by overlaying the “T-shaped” or the “Y-shaped” part of a structure of a full-length IgG1 (PDB ID: 1IGY) upon the Fc-YTE in the structure of FcRn in complex with Fc-YTE (PDB ID: 4N0U).

Several different binding models of IgG1s to FcRn have been proposed in the literature, where the two most prominent binding models are the “lying down” and the “standing-up” models^28,31,87^. In the “lying down” model, FcRn is positioned horizontally to the lipid membrane to accommodate binding of the full-length IgG, whereas in the “standing-up” model, FcRn is positioned perpendicular to the lipid membrane. The “lying down” model will inherently compromise the HSA binding site on FcRn and the possibility of obtaining avidity via IgG binding to two separate FcRn molecules^10^. On the other hand, the “standing-up” model requires the IgG1s to obtain the “T-shape” to avoid a steric clash with the lipid membrane. Furthermore, a third model has recently been proposed, the so-called “reclined model”, which is an intermediate between the two other models, which accommodates antigen binding to the Fab domains of the IgG (Fig. 4)^104^.

For all the proposed models, a direct interaction between the α3 domain of the FcRn molecule and the IgG Fab domains, as proposed in the current paper, is possible. However, it is important to note that the presented data in the current manuscript support a sequential two-pronged mechanism whereby the interaction between the Fc-part of IgG1s and FcRn precedes an interaction between the Fab domains and the α3 domain of FcRn^33^. For this mechanism to occur in the context of the endosomal membrane, the Fab domains would need to be pre-oriented toward the “T-shape” before FcRn binding can proceed (Fig. 4). We thus propose that rearrangement of IgG1 to the “T-shape” conformation occurs as part of the sequential mechanism involving first the Fc and subsequently the Fab domains binding to native membrane-tethered FcRn.

## Conclusions

We have used XL-MS and HDX-MS to confirm the canonical binding interface between FcRn residues in the α2 domain and β2m of FcRn, and residues in the Fc-part of IgG1s. Furthermore, XL-MS data identified a direct interaction between the Fab domains and several regions of FcRn, and HDX-MS identified changes in conformational dynamics in regions of the Fab domains upon FcRn binding. Similar XL-MS and HDX-MS results were obtained for both a human and a chimeric (mouse LC and human HC) IgG1 with different types of LCs (λ and κ). Hence, the interaction between the FcRn and the IgG Fab domains appears to be conserved and a common feature at least for IgG1 mAbs. Taken together, our results provide new molecular insights into a key interaction of the human immune system and could advance the development of therapeutic IgGs with tailored pharmacokinetics and enhanced drug efficacy.

## Methods

### Reagents

All reagents were purchased from Sigma-Aldrich in analytical grade, if the manufacturer is not defined, except the following reagents: TCEP (Thermo Scientific, USA), pepsin beads (Thermo Scientific, USA), DSS (D_0_/D_12_) (Creative Molecules Inc., Canada), LysC (Fujifilm Wako Pure Chemical Corp., Japan), PDH (D_0_) (abcr GmbH, Germany), AspN protease (Promega Corp., USA), and trypsin (Promega Corp., USA).

### Acquisition of mAbs

DNA vectors encoding the HC and LC of C-Mab were generated as previously described^40,105^. These vectors were used to transiently co-transfect human embryonic kidney (HEK) 293E cells at a 1:2 ratio of HC:LC encoding vectors, essentially as previously described^64^. HEK293E cells were cultivated in RPMI-1640 GlutaMAX medium supplemented with 25 U/ml penicillin and 25 μg/mL streptomycin. Growth medium was harvested and replaced every other day for two consecutive weeks following transfection. C-Mab was purified from the harvested medium by use of a CaptureSelect (Thermo Scientific, USA) affinity column with specificity for the CH1 domain of the C-Mab HC. Monomeric fractions were obtained by means of size-exclusion chromatography (SEC), where a Superdex 200 Increase 10/300 GL column (GE HealthCare, USA) was coupled to an Äkta FPLC instrument (GE HealthCare, USA), followed by up-concentration on Amicon Ultra-15 mL columns with a cut-off at 50K (Millipore, USA).

H-Mab was acquired from the pharmacy as the commercial product Humira™. Prior to its experimental use, H-Mab was buffer-exchanged into 1× sterile phosphate-buffered saline (PBS; Thermo Scientific, USA) by use of Amicon Ultra-15 mL columns with a cut-off at 50K. Following this, monomeric fractions were secured by SEC.

### Expression and purification of FcRn

Truncated, soluble human FcRn with a His-tag was produced with a Baculovirus expression system, as previously described^106,107^, by the use of a viral stock kindly gifted from Dr. Sally Ward (University of Southampton, UK). Briefly, FcRn was expressed in high-five cells, which were cultured at 1 × 10^6^ cells/mL at 27 °C and 160 rpm. 500 mL of this cell suspension was infected with 1 mL viral stock of *Autographica californica* nuclear polyhedrosis virus, which contained a pAxUW51 plasmid encoding the human β2m and α1-3 extracellular domains of FcRn fused to a C-terminal His_6_ tag. Following infection, the cells were cultured at 24 °C for 72 h, before harvesting the supernatant and adjusting it to pH 7.2. FcRn was purified by use of a HisTrap HP column containing Ni^2+^ ions (GE HealthCare, USA), as previously described^106^. Briefly, the column was equilibrated with PBS containing 0.05% sodium azide prior to application of the pH-adjusted supernatant. Following washing of the column with PBS and 25 mM imidazole, bound FcRn was eluted by application of 50 mL PBS containing 250 mM imidazole. Next, FcRn was up-concentrated by use of an Amicon Ultra-15 mL column with a cut-off at 10K (Millipore) and buffer-exchanged to PBS. Monomeric fractions of FcRn were obtained by running the resulting solution on SEC as described above for mAbs.

### HDX-MS of FcRn ± C-mAb or H-mAb, C-mAb ± FcRn, and H-mAb ± FcRn

Complex formation: The protein of interest (e.g., FcRn/C-mAb/H-mAb) was prepared alone or in the presence of a seven times molar excess of ligand (e.g., FcRn/C-mAb/H-mAb) in a phosphate/citric acid buffer (125.4 mM Na2HPO4, 37.3 mM citric acid), pH 6.0, at a concentration of 4 µM. In the presence of the ligand, we estimate that at least 81% of the protein of interest was ligand-bound under exchange conditions. The samples were incubated at 25 °C for 20 min before initiation of the exchange reaction to ensure equilibration. An overview of the analyzed samples and replicates is found in the Supplementary information (Supplementary Table S5). Exchange reaction: The exchange reaction was initiated by diluting the protein samples 1:9 into a deuterated buffer (125.4 mM Na_2_HPO_4_, 37.3 mM citric acid) pD_read_ 6.0. After various time intervals, the exchange reaction was quenched by diluting the sample 1:1 with ice-cold quench buffer (300 mM Phosphate Buffer pH 2.3, 6 M GndHCl, 0.5 M TCEP), decreasing the pH to 2.51. After quenching, the samples were quickly frozen to −80 °C and kept at −80 °C until analysis.

LC-MS analysis: The quenched samples were thawed and injected into a cooled (0 °C) reversed-phase UPLC-HDX-system (Waters). Here, the samples were first led through a home-packed pepsin column filled with pepsin beads. The resulting peptides were captured on a trap column (ACQUITY UPLC BEH C18 1.7 µm VanGuard column, Waters Inc., USA) and desalted for 3 min at 200 µL/min with Solvent A (0.23% formic acid in water). The trap column was put in-line with an C18 analytical column (ACQUITY UPLC BEH C18 1.7 µm, 1 × 100 mm column, Waters Inc.) and separated by traditional reversed-phase chromatography by a gradient of solvent A and solvent B (0.23% formic Acid in Acetonitrile) over 9 min and ionized by positive electrospray ionization into an ion mobility enabled Q-TOF mass spectrometer (Synapt G2-Si, Waters Inc., USA). Before final mass detection in the TOF, the peptides were separated according to their collision cross-section and charge in a traveling wave ion guide. Identification of peptides was performed on non-deuterated and fully reduced protein samples by MS/MS using both data-independent (MSe) and data-dependent acquisition.

Data analysis: For peptide identification, the acquired mass spectra (MS and MS/MS) were lock mass corrected against glu-1-fibrinopeptide B and analyzed in PLGS 3.0 (Waters Inc., USA), which matched precursor and fragment spectra with a local database consisting of FcRn, β2, IgG-H-mAb, IgG-C-mAb, pepsin and shuffled sequences hereof. All MS/MS spectra used for peptide identification were inspected and manually verified. For measuring the deuterium content of identified peptides following HDX, acquired mass spectra were lock mass corrected against glu-1-fibrinopeptide B and analyzed in DynamX 3.0 (Waters Inc., USA)

Statistics and Reproducibility of HDX-MS data: All statistical analyses were performed in Excel (Microsoft, USA). For data points recorded in triplicate, a comparative analysis was performed with either a heteroscedastic or homoscedastic Student’s T-test, depending on the equality of the variances of the compared data points. The equality of the variances was determined by performing an F-test with the significance level set to 0.05. A peptide was only considered to have experienced a significant change in HDX between two states if both of the following criteria were fulfilled^108,109^: (1) a significant difference in deuterium uptake (*p* < 0.01), and (2) the absolute difference in deuterium uptake should be larger than a global $\left|\triangle \overline{{HX}}\right|$ as defined by Hageman and Weis^109^. For data points, where replicate data were not recorded, statistical significance was determined by the use of only the latter criteria.

### XL-MS of FcRn in complex with mAbs

DSS cross-linking: 50 µg of protein complex (prepared either in a 1:1 or a 2:1 ratio between FcRn:mAb) was prepared in a concentration of 1 mg/mL in PBS, pH adjusted to pH 6.0. The protein sample was equilibrated for 20 min at 37 °C before the addition of 2 µL of 25 mM DSS (D_0_/D_12_) in anhydrous DMF. After 30 min the cross-linking reaction was quenched by the addition of 2.5 µL 1 M NH_4_HCO_3_.

PDH cross-linking^62^: 50 µg of protein complex (prepared either in a 1:1 or a 2:1 ratio between FcRn:mAb) was prepared in PBS, pH adjusted to pH 6.0. The protein sample was equilibrated for 20 min at 37 °C before the addition of 15.11 µL 27 mg/mL PDH (D_0_/D_10_) and 4.16 µL 144 mg/mL DMTMM. The final concentration of the protein complex was 1 mg/mL. After 30 min, the cross-linking reaction was stopped by spin filtering the solution through Zeba Spin Desalting Columns, 7 K MWCO (ThermoFischer, USA).

XPlex cross-linking^63^: 50 µg of protein complex (prepared either in a 1:1 or a 2:1 ratio between FcRn:mAb) was prepared in 200 mM MES buffer, pH adjusted to pH 6.0. The protein sample was equilibrated for 20 min at 25 °C before the addition of 2.53 µL 100 mg/mL hexanediamine (D_0_/D_12_), 8.62 µL 50 mg/mL EDC, and 11.75 µL 12.5 mg/mL HOBt. The final concentration of the protein complex was 1 mg/mL. After 30 min the cross-linking reaction was stopped by spin filtering the solution through Zeba Spin Desalting Columns, 7 K MWCO (ThermoFischer, USA).

All cross-linking reactions were performed at least in duplicate.

The results of the cross-linking reaction were followed by reducing and non-reducing SDS-PAGE as described below.

Proteolytic digestion: The cross-linked protein samples were evaporated to dryness in a vacuum centrifuge and re-suspended and reduced in 8M urea and 2.5 mM TCEP and incubated for 30 min at 37 °C in an Eppendorf Thermomixer (mixing speed 750 rpm). The samples were cooled to room temperature before a freshly prepared solution of iodoacetamide was added to a final concentration of 5 mM. To mitigate adverse reaction and inactivation of the iodoacetamide, the samples were kept in the dark for 30 min. For combined LysC and Trypsin digestion, the samples were diluted with 150 mM NH_4_HCO_3_ buffer to a urea concentration below 5 M before addition of LysC (1:100 w/w of substrate:enzyme) and incubated at 37 °C in for 2 h in an Eppendorf Thermomixer (mixing speed 750 rpm). Finally, the samples were diluted to 1 M urea by the addition of 50 mM NH_4_HCO_3_, trypsin was added (1:50 w/w of substrate:enzyme), and samples were incubated at 37 °C overnight in an Eppendorf Thermomixer (mixing speed 750 rpm). For AspN digestion, the samples were diluted to 1 M Urea by the addition of 50 mM NH_4_HCO_3_ buffer, and AspN was added (1:50 w/w of substrate:enzyme) and incubated at 37 °C overnight in an Eppendorf Thermomixer (mixing speed 750 rpm).

SPE clean-up: After overnight digestion, the samples were acidified with formic acid (2% final concentration) and purified by solid-phase extraction (Sep-Pak Vac 1cc (50 mg) t18 cartridges, Waters Inc., USA). The eluate (water/acetonitrile/formic acid, 50:50:0.1 v/v) was evaporated to dryness in a vacuum centrifuge.

SEC purification: The cross-linked peptide samples were re-suspended in 20 µL SEC running buffer (water/acetonitrile/trifluoroacetic acid, 70:30:0.1 v/v) and 15 µL were injected into a GE HealthCare Ӓkta micro system consisting of autosampler, binary pump, UV/pH/conductivity detectors, and fraction collector. Peptides were separated on a Superdex Peptide column (3.2 × 300 mm, GE HealthCare, USA) at a flow rate of 50 µL/min. The eluate was collected in a 96-well plate in fractions of 0.1 mL. Fractions of interest (e.g., 1.0–1.2 mL) were collected^53^, evaporated to dryness in a vacuum centrifuge, and re-suspended in LC solvent A (water/acetonitrile/formic acid, 95:5:0.1 v/v).

LC-MS analysis: Approximately 1 µg of each SEC fraction was loaded onto a nanoflow LC system (EASY-nLC 1000, ThermoFisher, USA) equipped with an Acclaim Pepmap column (150 mm × 75 μm, 2 μm particle size, 100 Å pore size, ThermoFisher, USA). The peptide mixture was separated by a 60 min gradient from 9% to 35% LC solvent B (0.1% formic acid in acetonitrile). The peptides were ionized by positive electrospray ionization into a hybrid ion trap-Orbitrap mass spectrometer (Orbitrap Elite, ThermoFischer, USA). The instrument was operated in DDA acquisition mode, where the survey scan was performed in the Orbitrap (350–1600 m/z) with a resolution of 120K. The 10 most abundant ions with a charge state ≥3 were fragmented by CID in the ion trap with 35% normalized collision energy. The mass of the resulting fragment ions was determined in the ion trap (200–2000 m/z). All SEC fractions were analyzed in technical duplicate.

Data analysis: The raw files of the MS/MS data were converted into the mzXML format by msconvert (ProteoWizard, USA)^110^. Cross-linked peptides were identified by xQuest (version 2.1.5)^111,112^. The search used a database containing the sequence of FcRn, β2m, the IgG of interest, and contaminants identified from a regular database search using Mascot and shuffled or reversed constructs of all proteins in the database^113^. The combination of the heavy and light scans was performed with the following settings: Precursor mass difference: 12.075321 Da (DSS and XPlex) or 10.06277 Da (PDH), retention time difference for light/heavy pairs: 1.0 min. For identification of cross-linked peptides containing a linker, the following settings were applied: Maximum number of missed cleavages (excluding the cross-linking site) = 2, enzyme specificities: Trypsin or AspN, peptide length = 4–40 amino acids, fixed modifications = carbamidomethyl-Cys (mass shift = 57.02146 Da), mass shift of the light cross-linker = 138.06808 Da (DSS), 152.10620 Da (PDH) or 80.11022 Da (XPlex), mass shift of mono-links = 155.09463 Da or 156.07864 Da (DSS), 170.11676 Da (PDH) or 98.12078 Da (XPlex). DSS was assumed to react with Lys residues or the N termini, PDH and XPlex were assumed to react with Asp and Glu residues, MS1 tolerance = 15 ppm, and MS2 tolerance = 0.2 Da for common ions and 0.3 Da for cross-link ions; the search was performed in ion tag mode. For identification of zero-length cross-links (PDH and XPlex treated samples), the following settings were applied: Maximum number of missed cleavages (excluding the cross-linking site) = 2, enzyme specificities: Trypsin or AspN, peptide length = 4–40 amino acids, fixed modifications = carbamidomethyl-Cys (mass shift = 57.02146 Da), mass shift of the cross-linker = −18.010595 Da (no mono-link mass was specified). DMTMM and XPlex were assumed to react with Asp and Lys or Glu and Lys residues. MS1 tolerance = 15 ppm, and MS2 tolerance = 0.2 Da for common ions and 0.3 Da for cross-link ions; the search was performed in enumeration mode.

Statistics and Reproducibility of XL-MS data: The cross-linked peptide candidates were post-filtered by applying a precursor mass window of ±5 ppm, and the false discovery rate (FDR) was adjusted to less than 0.05 at the non-redundant peptide pair level by the xProphet software^112^.

Only identifications of cross-linked peptide pairs with an ld-score ≥25 or an FDR <0.05, n-seen ≥2, and observed in at least two experimental replicates or conditions were regarded as hits. A list of XLs not surviving these criteria, but with an ld-score ≥25 or an FDR <0.05, can be found in the supplemental information (Supplementary Table S3).

### SDS-PAGE

5 µg of protein material was evaporated to dryness in a vacuum centrifuge and re-suspended in NuPAGE™ LDS Sample Buffer (Invitrogen, USA). For selected samples, the sample buffer was also supplemented with 10 mM TCEP. The SDS-PAGE samples were heated for 10 min at 70 °C before loading onto 1.5 mm NuPAGE 4–12% Bis-Tris protein gels (Invitrogen, USA). Electrophoresis was performed for 50 min at 200 V in MOPS buffer (Thermo Scientific). The gels were stained with Simple blue staining buffer (Thermo Scientific) and destained with demineralized water.

### XL-MS control experiments

Cross-linking of C-mAb with β2m: C-mAb in the presence of a two-fold molar excess of β2m without FcRn was cross-linked. A similar procedure was followed as described for cross-linking of FcRn:IgG complexes. Both LysC/trypsin and AspN digestions were performed.

IdeZ treatment of IgG: 50 µg of IgG (5 mg/mL) was added 1 µL of IdeZ solution (Promega Corp., USA). The mixture was incubated at 37 °C for 30 min to enable cleavage of the Fab and Fc region of the IgG. The reaction result was followed by SDS-PAGE (Supplementary Fig. S16). After confirmation of successful cleavage of the Fc from the Fab parts by SDS-PAGE FcRn was added in a 1:2 molar ratio, and the samples were treated as described above for DSS XL-MS of FcRn in complex with mAbs.

### Interaction space analysis

The interaction space analysis was performed on the DisVis Server (https://wenmr.science.uu.nl/disvis/)^68,69^. The crystal structures of FcRn (PDB ID: 4N0U)^25^ and of H-mAb Fab-fragment (PDB ID: 4NYL) or the C-mAb Fab-fragment (PDB ID: 1NGP)^41^, were used as the fixed chain and the scanning chains, respectively. The distance restraint of DSS was set to 30 Å.

### Homology model of H-mAb and C-mAb

The homology models of H-mAb and C-mAb were built in the SWISS-MODEL server (https://swissmodel.expasy.org/interactive)^114,115^ with the crystal structure (PDB ID: 1IGY)^58^ as the template.

### Reporting summary

Further information on research design is available in the Nature Portfolio Reporting Summary linked to this article.

## Supplementary information

Supplementary Information

Reporting Summary

## Data Availability

The mass spectrometry data and numeric source data from the XL-MS experiments have been deposited to the ProteomeXchange Consortium via the PRIDE partner repository with the data set identifier PXD044405^116^. The mass spectrometry data and numeric source data from the HDX-MS experiments have been deposited to the ProteomeXchange Consortium via the PRIDE partner repository with the data set identifier PXD044285^116^. All other data are available from the corresponding author upon reasonable request.
