# Supplementary material for: Identification of a direct interaction between the Fab domains of IgG antibodies and human FcRn upon IgG-FcRn complex formation
Source: Commun Biol. 2025 Jun 12;8:922. doi: 10.1038/s42003-025-08252-z (PMC12162884; doi:10.1038/s42003-025-08252-z)
Supplement: Supplementary file 1 — Supplementary Information [file 42003_2025_8252_MOESM1_ESM.pdf]

Supplemental Information

Identification of a direct interaction between the Fab domains of IgG antibodies and human FcRn upon IgG-FcRn complex formation

Esben Trabjerg[1,#], Jeannette Nilsen[3,4,5], Torleif Tollefsrud Gjølberg[3,4,5], Jan Terje Andersen[3,4,5], Alexander Leitner[1] and Kasper D. Rand[2,*]

[1]Department of Biology, Institute of Molecular Systems Biology, ETH Zürich, Zurich, Switzerland

[2]Department of Pharmacy, University of Copenhagen, Copenhagen, Denmark

[3]Precision Immunotherapy Alliance (PRIMA), University of Oslo, Oslo, Norway

[4]Department of Immunology, Oslo University Hospital Rikshospitalet and University of Oslo, Oslo, Norway

[5]Institute of Clinical Medicine, Department of Pharmacology, University of Oslo and Oslo University Hospital, Oslo, Norway

[#]current address: Department of Pharmacy, University of Copenhagen, Copenhagen, Denmark

*To whom correspondence should be addressed.

Table of Contents

Page S-3: **Supplemental Methods**

Page S-4: **Table S1 –** Table of identified cross-links, including overview of identified fragment ions.

Page S-6: **Table S2** – Identification of overlength cross-links between Fab domains and the Fc part of C-mAb.

Page S-7: **Table S3 –** Cross-links only observed in a single experimental condition or only observed in the FcRn:mutC-mAb complex.

Page S-8: **Table S4** – Identified inter protein cross-links bewteen IdeZ-treated mAbs and FcRn

Page S-9: **Table S5 –** Oveview of recorded HDX data.

Page S-10: **Figure S1** – Alignment of HCs of H-mAb with C-mAb and HC of H-mAb with Fc-YTE.

Page S-11: **Figure S2** – Alignment of LCs of H-mAb and C-mAb.

Page S-12: **Figure S3** – Charge distribution and pH-dependent net charge of the Fab domains of H-mAb and C-mAb.

Page S-13: **Figure S4** – Conformational flexibility of full-length IgGs.

Page S-14: **Figure S5** – Visualization of cross-links identified in the complex between mutC-mAb and FcRn.

Page S15: **Figure S6** – Observation of higher order oligomers after cross-linking a solution of FcRn and C-mAb

Page S-16: **Figure S7** – DisVis analysis of mAbs Fab domain interaction with FcRn.

Page S-17: **Figure S8** – Effective HDX-MS coverage map of H-mAb.

Page S-18: **Figure S9** – Effective HDX-MS coverage map of C-mAb.

Page S-19: **Figure S10 –** HDX plots of H-mAb in the absence and presence of FcRn.

Page S-23: **Figure S11 –** HDX plots of C-mAb in the absence and presence of FcRn.

Page S-26: **Figure S12 –** Effective HDX-MS coverage map of FcRn.

Page S-27 **Figure S13 –** HDX plots of FcRn in the absence and presence of either H-mAb or C-mAb (exchange time: 2.09 min to 1507.13 min).

Page S-29: **Figure S14** - Conformational response of the complex formation of FcRn and C-mAb.

Page S-30: **Figure S15** – HDX plots of FcRn in the absence and presence of either H-mAb or C-mAb (exchange time: 13 s to 2.09 min).

Page S-33: **Figure S16 –** SDS-PAGE of IdeZ treated C-mAb.

Page S-34: **Supplemental References**

Supplemental Methods

Calculation and visualization of surface charge: Surface charge analysis was done based on the crystal structures of the Fab domains of H-mAb and C-mAb (PDB ID: 4NYL and 1NGP). The surface charge maps were generated by the pdb2pqr (3.6.1) and APBS (3.4.1) servers (https://server.poissonboltzmann.org/pdb2pqr) with standard settings. The protonation states of side chains were calculated by the PROPKA algorithm and the PARSE forcefield was used. The resulting surface potential maps where visualised at $\pm 3k_BT/e$ in PyMOL (2.5.2). The sequence based pH-dependent net charge of the Fab domains of H-mAb and C-mAb were calculated using the Emboss iep calculator (http://www.bioinformatics.nl/cgi-bin/emboss/iep?_pref_hide_optional=0).[1] All cysteines were assumed to form disulfide bridges and the Fab domiains were set to contain 2 N-terminals and 1 C-termini.

In-gel digestion of C-mAb: The SDS-PAGE band corresponding to monomeric C-mAb was sliced of the gel, cut into smaller pieces, placed in a Eppendorf tube, solvated with 200µL 25 mM 25mM NH_4HCO_3 in 50:50 MQ water:acetonitrile, and incubated at 37 ºC under shaking for 30 min. The supernatant was discarded and the process was repeated twice to de-stain the gel pieces. The gel pieces were dehydrated with 100µL acetonitrile, washed with 100µL MQ water, dehydrated with 100µL acetonitrile before addition of 100µL 6.5 mM dithiothreitol in MQ water and incubated at 60 ºC under shaking for 60 min to ensure efficient reduction of C-mAb. The solvent was discarded and the gel pieces were dehydrated in 100µL acetonitrile before addition of 100µL 54 mM iodoacetamide in MQ water and incubated at 25 ºC in the dark under shaking for 30 min to alkylate C-mAb. The solvent was discarded and the gel pieces was dehydrated with 100µL acetonitrile and hydrated with 50mM NH_4HCO_3 in MQ water. This process was repeated 3 times, and the gel pieces were dehydrated a final time in 100µL acetonitrile. The supernatant was discarded and the dehydrated gel pieces were hydrated in 100µL of 10ng/µL trypsin solution in 50mM NH_4HCO_3. The gel pieces were incubated overnight at 37 ºC under shaking. The next morning the supernatant was collected. The gel pieces were covered in 40µL 0.1 % formic acid in MQ water and incubated for 15 min at room temperature under shaking. The supernatant was collected. The gel pieces were covered in 40µL of a 50:50 mixture of 0.1 % formic acid in MQ water and acetonitrile and incubated for 15 min at room temperature under shaking. The supernatant was collected. The gel pieces were covered in 40µL acetonitrile and incubated for 15 min at room temperature under shaking. All collected supernatants were pooled and evaporated to dryness in a vacuum centrifuge. The samples were re-suspended in 20µL SEC running buffer, purified by SEC and analyzed by LC-MS as described for XL-MS samples in the methods section.

Supporting Table S1. Table of identified cross-links including overview of identified fragment ions. A “Z”-residue denotes the end amino group on the N-terminal of a protein chain (cross-link number 8).

	Residue 1	Residue 2	Peptide 1	Peptide 2	Id-Score	Euclidean Cα-Cα distance (Å)	Identified fragments ions<br>Red: fragment ion containing cross-linker,<br>Green: Fragment ions not containing cross-linker
**1**	β2m - K91	Fc HC - K292	VNHVTLSQP**K**IVK	FNWYVDGVEVHNAKTKPR	34.11	16.8	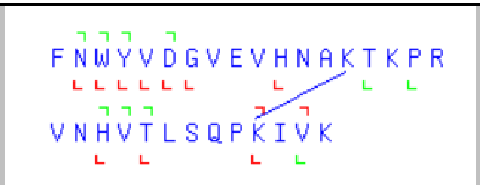
**2**	β2m - K91	Fc HC - K294	VNHVTLSQPKIVK	FNWYVDGVEVHNAKTKPR	36.36	20.5	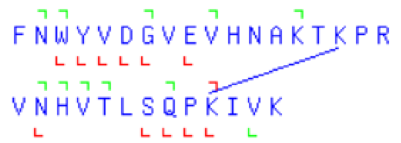
**3**	β2m - K6	Fc HC - K292	TPKIQVYSR	FNWYVDGVEVHNAKTKPR	33.26	24.0	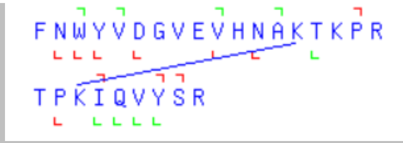
**4**	α3 - K243	Fab HC - K217	DGSFHASSSLTVKSG	DKKVEPKSC	36.16		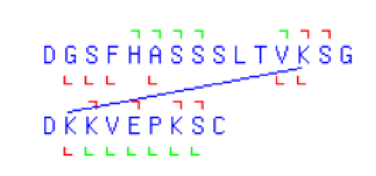

**5**	α3 - K243	Fab HC - K218	DGSFHASSSLTVKSG	DKKVEPKSC	40.56	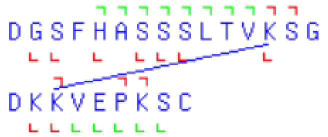
**6**	β2m - K91	Fab HC - K59	VNHVTLSQPKIVK	IDPNSGGTKYNEK	33.41	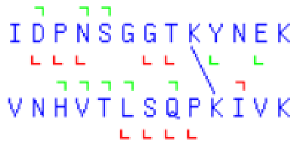
**7**	β2m - K94	Fab HC – K59	IVKWDRDM	IDPNSGGTKYNEK	37.86	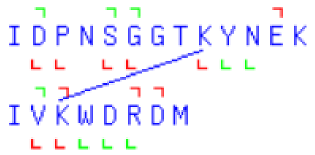
**8**	β2m - K91	Fab HC – E1 (N-term)	VNHVTLSQPKIVK	EVQLVESGGGLVQPGR	32.60	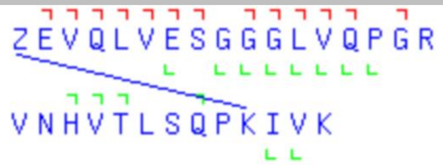

Supporting Table S2. Identification of overlength cross-links between Fab domains and the Fc part of C-mAb. C-mAb was cross-linked with DSS as described in the methods section. After cross-linking the cross-linking products where seperatated on an SDS-PAGE as described in the methods section. The band corresponding to monomeric C-mAb was sliced out of the gel and in-gel digestion with LysC and trypsin was performed, see supporting methods. The released peptides were analysed by LC-MS as described in the methods section. 5 overlenght cross-links where identified highlighting the flexibility of the Fab domains in C-mAb. The Euclidan distances where determined in Pymol on the homology model of C-mAb. The highest Id-score observed across all data sets is shown.

Residue 1	Residue 2	Id-Score	Replicates	Euclidean Cα-Cα distance (Å)
non overlength XL				
HC - 137	LC - 207	25.19	1/2	11.1
HC - 137	HC - 218	36.4	2/2	19.9
HC - 137	HC - 222	28.98	2/2	10.6
HC - 218	LC - 207	32.8	1/2	24.5
HC - 364	HC - 418	39.9	2/2	7.8
Overlength XL				
HC - 59	HC - 344	31.75	1/2	88.3
HC - 59	HC - 418	37.59	2/2	79.5
HC - 137	HC - 330	32.54	2/2	39.8
LC - 207	HC - 344	37.94	1/2	60.0
HC - 63	HC - 418	32.22	1/2	76.0

Supporting Table S3. Cross-links only observed in a single experimental condition or only observed in the FcRn:mutC-mAb complex. The abbreviation in the "Residue 1 and 2" columns refers to the domains of FcRn (α1, α2, α3 or β2m) and IgGs (LC or HC). The "Complex" column refers to in which complex the cross-link have been identified. The "Chemistry" column refers to the cross-linking chemistry applied. The "Enzymes" column refers to the used proteases. The "Id-Score / FDR" refers to the Id-score in xQuest, while the FDR was calculted in xProphet. The "n-seen" column refers to number of spectrums, where the cross-link was identified.

	Residue 1	Residue 2	Complex	Chemistry	Enzymes	Id-Score / FDR	n-seen
**1**	β2m - 6	HC - 292/294	FcRn:H-mAb	DSS	LysC + Tryp	33.26	1
**2**	α2 - 146	HC - 76	FcRn:H-mAb	DSS	LysC + Tryp	40.74	3
**3**	β2m - 91	HC - 418	FcRn:H-mAb, FcRn:C-mAb, FcRn:mutC-mAb	DSS	LysC + Tryp	36.08	1
**4**	β2m - 91	LC - 145	FcRn:H-mAb	DSS	LysC + Tryp	26.16	1
**5**	α2 - 146	HC - 418	FcRn:H-mAb	DSS	LysC + Tryp	26.83	1
**6**	α2 - 146	LC - 1	FcRn:H-mAb	DSS	LysC + Tryp	37.38	1
**7**	β2m - 91	LC - 107	FcRn:H-mAb	DSS	LysC + Tryp	37.4	1
**8**	β2m - 91	LC - 188	FcRn:H-mAb	DSS	LysC + Tryp	39.09	1
**9**	α2 - 177	LC - 1	FcRn:H-mAb	DSS	LysC + Tryp	30.76	1
**10**	α1 - 59	HC - 74	FcRn:C-mAb	DSS	LysC + Tryp	25.11	2
**11**	β2m - 91	HC - 278	FcRn:H-mAb	DSS	AspN	36.12	3
**12**	β2m - 41	HC - 343	FcRn:H-mAb	DSS	AspN	25.63	1
**13**	β2m - 94	HC - 278	FcRn:H-mAb	DSS	AspN	27.71	1
**14**	β2m - 94	HC - 46	FcRn:H-mAb	PDH-ZL	LysC + Tryp	23.24 / 0	2
**15**	α2 - 146	LC - 207	FcRn:mutC-mAb	DSS	LysC + Tryp	29.28	1
**16**	β2m - 48	LC - 72	FcRn:mutC-mAb	DSS	AspN	28.16	1
**17**	β2m - 91	HC - 217/218	FcRn:mutC-mAb	DSS	AspN	27.01	1
**18**	α1 - 67	LC - 206	FcRn:mutC-mAb	XP	LysC + Tryp	37.77	1
**19**	α2 - 168	LC - 163	FcRn:mutC-mAb	XP	LysC + Tryp	36.52	1
**20**	α2 - 178	LC71	FcRn:mutC-mAb	XP	LysC + Tryp	27.43	1
**21**	β2m - 77	HC - 46	FcRn:mutC-mAb	XP	LysC + Tryp	37.93	1
**22**	α1 - 54/62	HC - 237	FcRn:mutC-mAb	XP	AspN	32.05	2
**23**	β2m - 38	HC - 262/269	FcRn:mutC-mAb	PDH	LysC + Tryp	29.43	2
**24**	α1 - 71	LC -71	FcRn:mutC-mAb	XP-ZL	LysC + Tryp	40.88 / 0	2
**25**	β2m - 50/53	HC - 217/218	FcRn:mutC-mAb	XP-ZL	LysC + Tryp	37.36 / 0	1
**26**	β2m - 50	HC - 330	FcRn:mutC-mAb	XP-ZL	LysC + Tryp	37.29 / 0	1
**27**	β2m - 53	HC - 330	FcRn:mutC-mAb	XP-ZL	LysC + Tryp	33.76 / 0	1
**28**	β2m - 48	LC - 206	FcRn:mutC-mAb	XP-ZL	LysC + Tryp	33.56 / 0	1
**29**	β2m - 94	LC - 201/206	FcRn:mutC-mAb	XP-ZL	LysC + Tryp	31.95 / 0	2
**30**	α1 - 71	LC - 80	FcRn:mutC-mAb	XP-ZL	LysC + Tryp	31.57 / 0	1
**31**	α2 - 178	HC - 418	FcRn:mutC-mAb	XP-ZL	LysC + Tryp	30.09 / 0.05	2
**32**	α2 - 145	HC - 67	FcRn:mutC-mAb	XP-ZL	LysC + Tryp	26.89 / 0	1
**33**	α2 - 165	HC - 330	FcRn:mutC-mAb	XP-ZL	LysC + Tryp	31.24 / 0	3
**34**	β2m - 50	HC 324/326	FcRn:mutC-mAb	PDH-ZL	LysC + Tryp	24.37 / 0	4

Supporting Table S4. Identified inter protein cross-links bewteen IdeZ-treated mAbs and FcRn. The identified cross-links below all belong to the IdeZ treated mutC-mAb in complex with FcRn, confirming the experimental conditions. As no interprotein cross-links have been identified between the Fc-part of an mAb and FcRn in a AspN digest in the current study this is expected. The Euclidan distances where determined in Pymol on the homology model of C-mAb. The highest Id-score observed across all data sets is shown

	Residue 1	Residue 2	Id-Score	Euclidean Cα-Cα distance (Å)
**1**	β2m - K91	Fc HC - K292	34.37	16.8
**2**	β2m - K91	Fc HC - K294	36.59	20.5
**3**	β2m - K6	Fc HC - K294	35.79	24.0

Supporting Table S5. Overview of recorded HDX data. The numbers in the table refers to the number of replicate(s) of recorded HDX data for every single time point at every condition. N/A: Not applicable.

Experiment	Protein	Ligand	Molar Excess of Ligand	13 s	25 s	38 s	2.09 min	6min 17s	25min 7s	3t 25min 45s	4t 11min 11s	1d 1t 7min 8s	Maximally labelled samples
	FcRn	Not present	N/A				3	3	3		3	3	3
I	FcRn	H-mAb	7 times				3	1	1		1	1	
	FcRn	C-mAb	7 times				3	3	3		3	3	
	FcRn	Not present	N/A	3	3	3	3						3
II	FcRn	H-mAb	7 times	3	3	3	3						
	FcRn	C-mAb	7 times	3	3	3	3						
III	H-mAb	Not present	N/A				3	1	1	1			1
	H-mAb	FcRn	7 times				3	1	1	1			
IV	C-mAb	Not present	N/A				3	3	3		3	3	
	C-mAb	FcRn	7 times				3	3	3		3	3	3

A)

```
Identities:391/451(87%), Positives:415/451(92%), Gaps:1/451(0%)

H-mAb  1    EVQLVESGGGLVQPGRSLRLSCAASGFTFDDYAMHWVRQAPGKGLEWVSAITWNSGHIDY  60
            +VQL + G  LV+PG S++LSC ASG+TF  Y MHWV+Q PG+GLEW+  I  NSG   Y
C-mAb  1    QVQLQQPGAELVKPGASVKLSCKASGYTFTSYWMHWVKQRPGRGLEWIGRIDPNSGGTKY  60

H-mAb  61   ADSVEGRFTISRDNAKNSLYLQMNSLRAEDTAVYYCAKVSYLSTASSLDYWGQGTLVTVS  120
             +  + + T++ D   ++ Y+Q++SL +ED+AVYYCA+  Y   +S  DYWGQGT +TVS
C-mAb  61   NEKFKSKATLTVDKPSSTAYMQLSSLTSEDSAVYYCARYDYYG-SSYFDYWGQGTTLTVS  120

H-mAb  121  SASTKGPSVFPLAPSSKSTSGGTAALGCLVKDYFPEPVTVSWNSGALTSGVHTFPAVLQS  180
            SASTKGPSVFPLAPSSKSTSGGTAALGCLVKDYFPEPVTVSWNSGALTSGVHTFPAVLQS
C-mAb  121  SASTKGPSVFPLAPSSKSTSGGTAALGCLVKDYFPEPVTVSWNSGALTSGVHTFPAVLQS  180

H-mAb  181  SGLYSLSSVVTVPSSSLGTQTYICNVNHKPSNTKVDKKVEPKSCDKTHTCPPCPAPELLG  240
            SGLYSLSSVVTVPSSSLGTQTYICNVNHKPSNTKVDKKVEPKSCDKTHTCPPCPAPELLG
C-mAb  181  SGLYSLSSVVTVPSSSLGTQTYICNVNHKPSNTKVDKKVEPKSCDKTHTCPPCPAPELLG  240

H-mAb  241  GPSVFLFPPKPKDTLMISRTPEVTCVVVDVSHEDPEVKFNWYVDGVEVHNAKTKPREEQY  300
            GPSVFLFPPKPKDTLMISRTPEVTCVVVDVSHEDPEVKFNWYVDGVEVHNAKTKPREEQY
C-mAb  241  GPSVFLFPPKPKDTLMISRTPEVTCVVVDVSHEDPEVKFNWYVDGVEVHNAKTKPREEQY  300

H-mAb  301  NSTYRVVSVLTVLHQDWLNGKEYKCKVSNKALPAPIEKTISKAKGQPREPQVYTLPPSRD  360
            NSTYRVVSVLTVLHQDWLNGKEYKCKVSNKALPAPIEKTISKAKGQPREPQVYTLPPSRD
C-mAb  301  NSTYRVVSVLTVLHQDWLNGKEYKCKVSNKALPAPIEKTISKAKGQPREPQVYTLPPSRD  360

H-mAb  361  ELTKNQVSLTCLVKGFYPSDIAVEWESNGQPENNYKTTPPVLDSDGSFFLYSKLTVDKSR  420
            ELTKNQVSLTCLVKGFYPSDIAVEWESNGQPENNYKTTPPVLDSDGSFFLYSKLTVDKSR
C-mAb  361  ELTKNQVSLTCLVKGFYPSDIAVEWESNGQPENNYKTTPPVLDSDGSFFLYSKLTVDKSR  420

H-mAb  421  WQQGNVFSCSVMHEALHNHYTQKSLSLSPG  450
            WQQGNVFSCSVMHEALHNHYTQKSLSLSPG
C-mAb  421  WQQGNVFSCSVMHEALHNHYTQKSLSLSPG  450
```

B)

```
Identities:206/209(99%), Positives:207/209(99%), Gaps:0/209(0%)

H-mAb  240  GGPSVFLFPPKPKDTLMISRTPEVTCVVVDVSHEDPEVKFNWYVDGVEVHNAKTKPREEQ  299
            GGPSVFLFPPKPKDTL I+R PEVTCVVVDVSHEDPEVKFNWYVDGVEVHNAKTKPREEQ
Fc-YTE 236  GGPSVFLFPPKPKDTLYITREPEVTCVVVDVSHEDPEVKFNWYVDGVEVHNAKTKPREEQ  295

H-mAb  300  YNSTYRVVSVLTVLHQDWLNGKEYKCKVSNKALPAPIEKTISKAKGQPREPQVYTLPPSR  359
            YNSTYRVVSVLTVLHQDWLNGKEYKCKVSNKALPAPIEKTISKAKGQPREPQVYTLPPSR
Fc-YTE 296  YNSTYRVVSVLTVLHQDWLNGKEYKCKVSNKALPAPIEKTISKAKGQPREPQVYTLPPSR  355

H-mAb  360  DELTKNQVSLTCLVKGFYPSDIAVEWESNGQPENNYKTTPPVLDSDGSFFLYSKLTVDKS  419
            DELTKNQVSLTCLVKGFYPSDIAVEWESNGQPENNYKTTPPVLDSDGSFFLYSKLTVDKS
Fc-YTE 356  DELTKNQVSLTCLVKGFYPSDIAVEWESNGQPENNYKTTPPVLDSDGSFFLYSKLTVDKS  415

H-mAb  420  RWQQGNVFSCSVMHEALHNHYTQKSLSLS  447
            RWQQGNVFSCSVMHEALHNHYTQKSLSLS
Fc-YTE 416  RWQQGNVFSCSVMHEALHNHYTQKSLSLS  444
```

Supporting Figure S1. Alignment of HCs of H-mAb with C-mAb (A) and HC of H-mAb with Fc-YTE (B). Residues marked in bold are directly implicated in the binding of Fc-YTE to FcRn according to the crystal structure solved by Oganesyan et al..[2] The alignment was performed by the BLAST algorithm.[3,4]

```
Identities:87/215(40%), Positives:123/215(57%), Gaps:11/215(5%)

H-mAb  1    DIQMTQSPSSLSASVGDRVTITCRASQG---IRNYLAWYQQKPGKAPKLLIYAASTLQSG  57
                    S+L+ S G+ VT+TCR+S G     NY  W Q+KP      LI   +    G
C-mAb  1    -QAVVTQESALTTSPGETVTLTCRSSTGAVTTSNYANWVQEKPDHLFTGLIGGTNNRAPG  59

H-mAb  58   VPSRFSGSGSGTDFTLTISSLQPEDVATYYCQRYNRAPYTFGQGTKVEI-KRTVAAPSVF  116
            VP+RFSGS  G    LTI+  Q ED A Y+C  +    + FG GTK+ +  +  ++PSV
C-mAb  60   VPARFSGSLIGDKAALTITGTQTEDEAMYFCALWYSNHWVFGGGTKLTVLGQPKSSPSVT  119

H-mAb  117  IFPPSDEQLKSGTASVVCLLNNFYPREAKVQWKVDNALQSGNSQESVTEQDSKDST--YS  174
            +FPPS E+L++  A++VC + +FYP    V WKVD    +  +Q   T Q SK S   Y
C-mAb  120  LFPPSSEELETNKATLVCTITDFYPGVVTVDWKVDG---TPVTQGMETTQPSKQSNNKYM  176

H-mAb  175  LSSTLTLSKADYEKHKVYACEVTHQGLSSPVTKSFNRGEC  214
             SS LTL+   +E+H  Y+C+VTH+G +  V KS +R +C
C-mAb  177  ASSYLTLTARAWERHSSYSCQVTHEGHT--VEKSLSRADC  215
```

Supporting Figure S2. Alignment of LCs of H-mAb and C-mAb. The alignment was performed by the BLAST algorithm.[3,4]

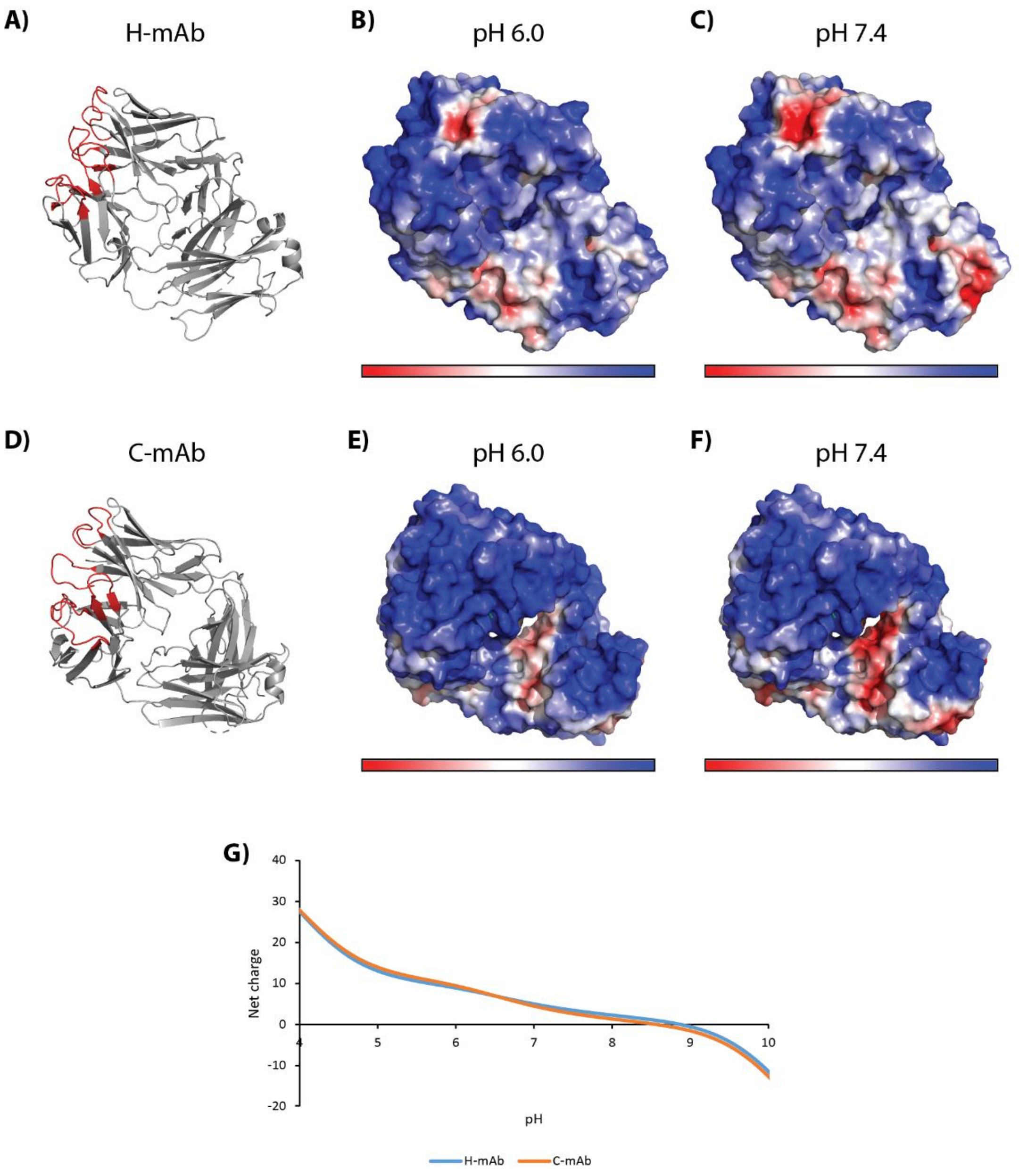

Supporting Figure S3. Charge distribution and pH-dependent net charge of the Fab domains of H-mAb and C-mAb. Crystal structure of the Fab domains of H-mAb (A) and C-mAb (D) (PDB ID:4NYL and 1NGP). CDRs are highlighted in red. B-C) Isoelectrical surface maps of H-mAb at pH 6.0 and pH 7.4 contured at ±3k_BT/e, red; negative and blue: positive. E-F) Isoelectrical surface maps of C-mAb at pH 6.0 and pH 7.4 contured at ±3k_BT/e, red; negative and blue: positive. G) pH-dependent sequence based net charge of the Fab domains of H-mAb (*blue* line) and C-mAb (*orange* line). See supplemental methods for further details.

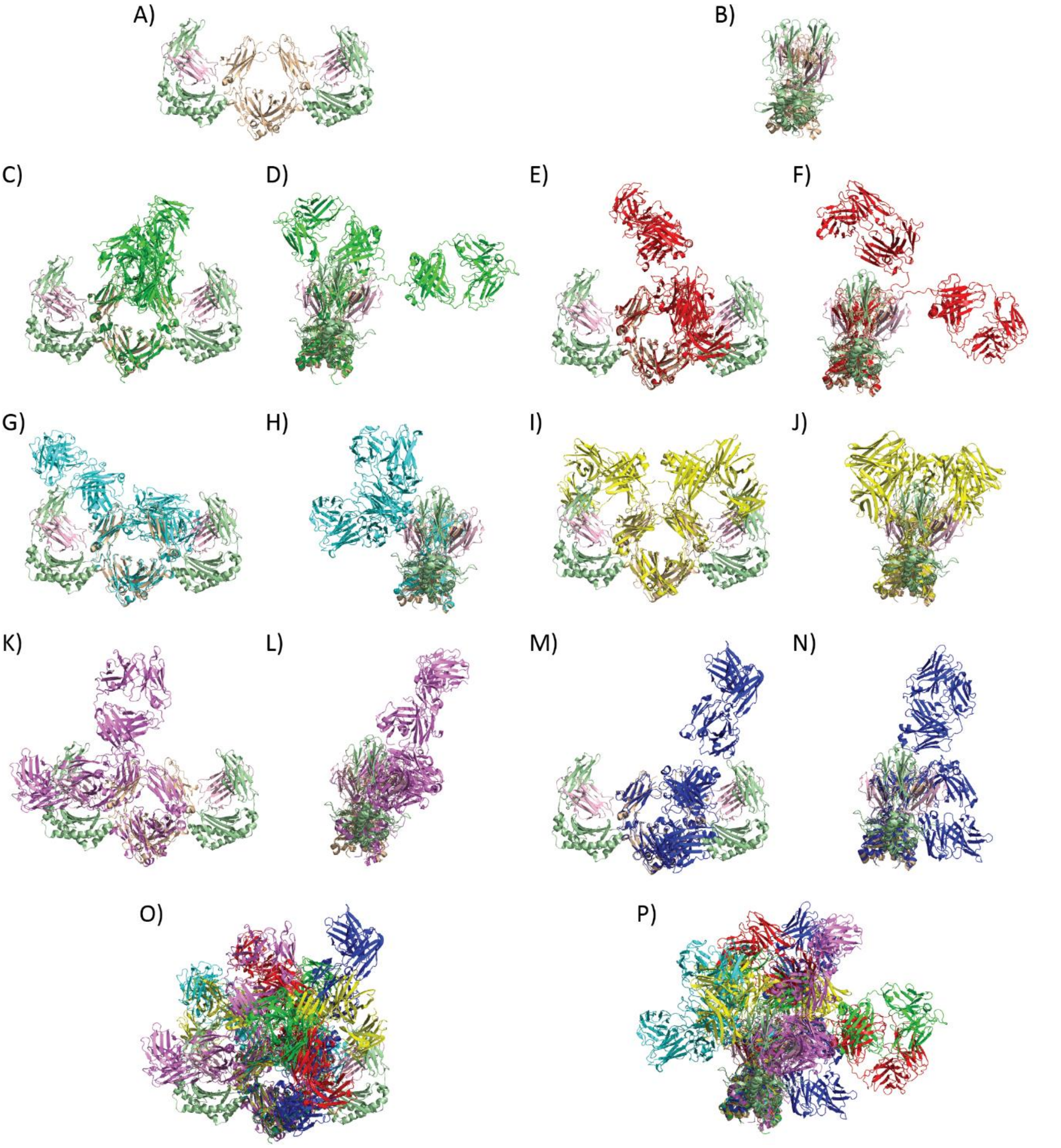

Supporting Figure S4: Conformational flexibility of full-length IgGs. Six crystal structures of full-length IgGs (PDB ID: 1IGT, 1HZH, 6GFE, 1MCO, 5FK3, and 1IGY) were aligned with the Fc part of the crystal structure of Fc-YTE in complex with FcRn (PDB ID: 4N0U, human serum albumin was omitted to simplify the figure).A-B) FcRn in complex with Fc-YTE (PDB ID: 4N0U) C-N) Individual IgGs shown with FcRn in complex with Fc-YTE. O-P) All IgG structures are shown at the same time. *Red*: 1IGT, *green*: 1HZH, *blue*: 6GFE, *yellow*: 1MCO, *violet*: 5DK3, *cyan*: 1IGY, *pale green*: FcRn, *light pink*: β-2-microglobulin, *wheat*: Fc-YTE. All figures are presented in two different views: a front view (A, C, E, G, I, K, M and O) and a view where the front view is rotated 90° counter clockwise along the horizontal axis (B, D, F, H, J, L, N and P).

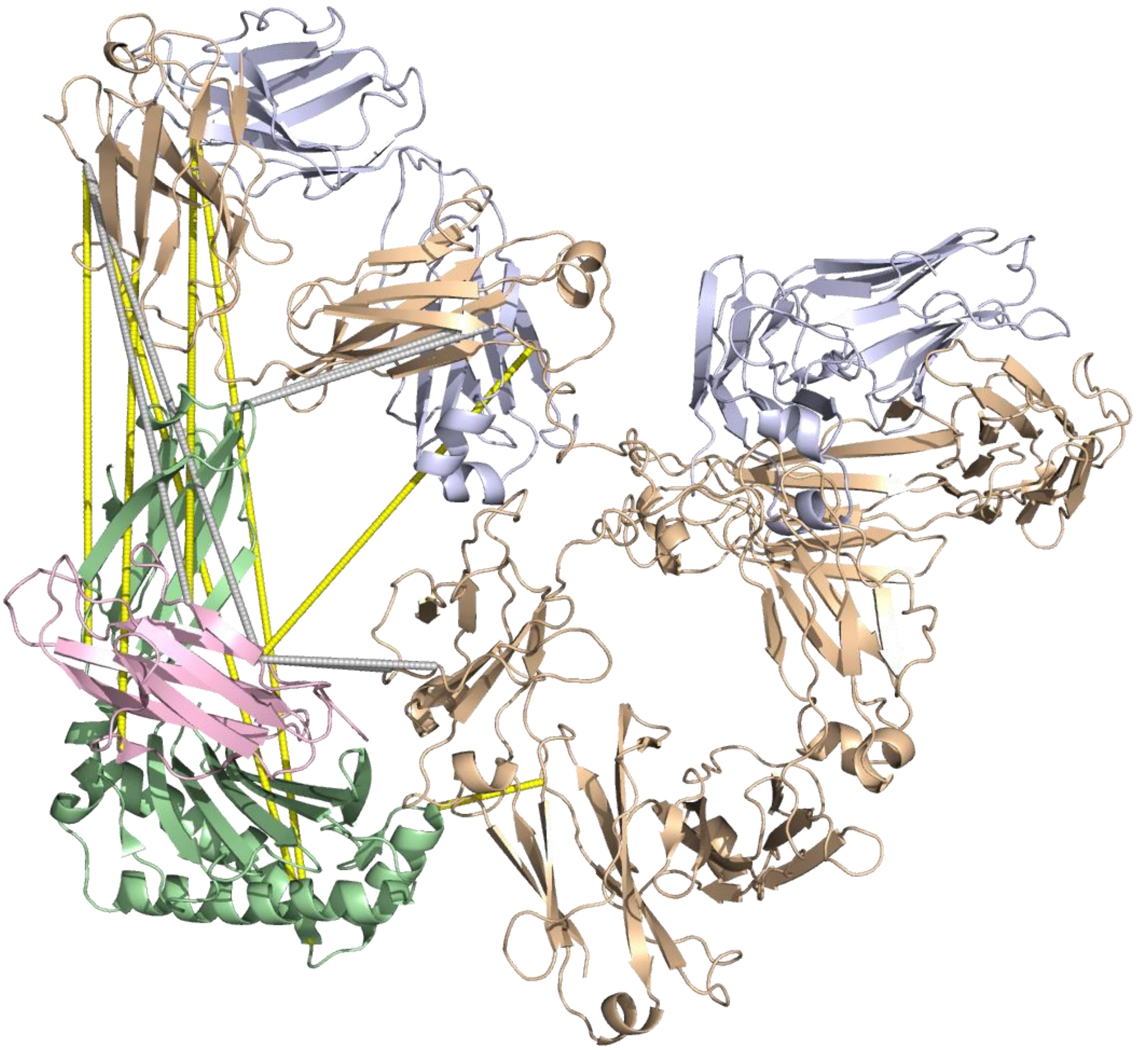

Supporting Figure S5: Visualization of cross-links identified in the complex between mutC-mAb and FcRn. Cross-links also found in the complex of C-mAb and FcRn are colored gray, while the cross-links only identified in the mutC-mAb:FcRn complex are colored yellow. The cross-links between mutC-mAb and FcRn illustrates several different binding modes not in line with the published crystal structure of FcRn:Fc-YTE complex (PDB ID: 4N0U). *Pale green*: α domains of FcRn, *light pink*: β2m, *wheat*: HC of mutC-mAb, *light blue*: LC of mutC-mAb.

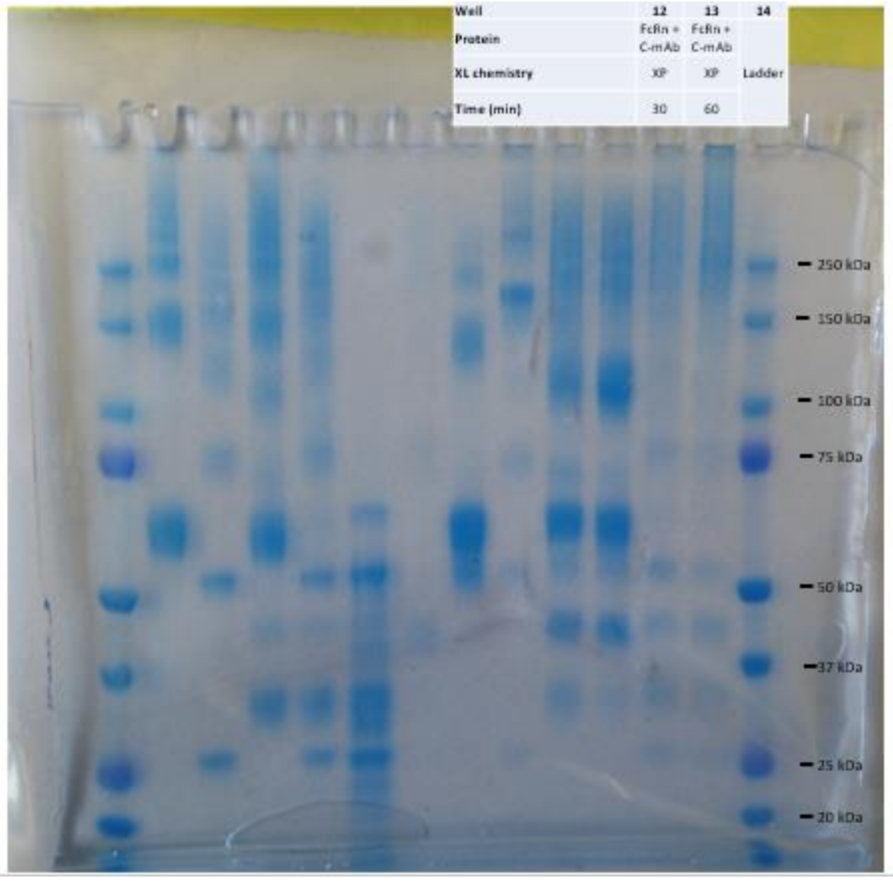

Supporting Figure S6: Observation of higher order oligomers after cross-linking a solution of FcRn and C-mAb with XPlex cross-linking (XP).

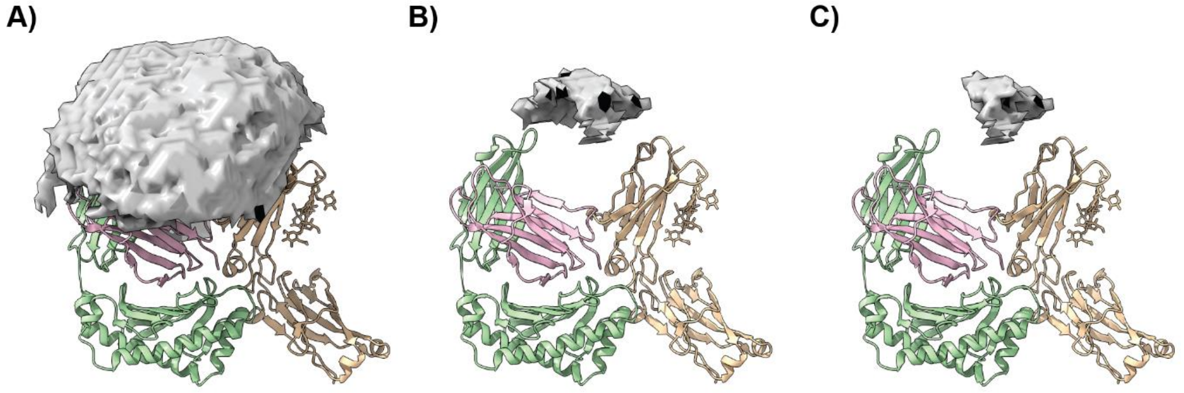

Supporting Figure S7: DisVis analysis of mAbs Fab domain interaction with FcRn. A) Interaction space of the Fab domain of H-mAb defined by the identified cross-links between H-mAb Fab domains and FcRn. B) Interaction space of the C-mAb Fab domain defined by the identified cross-links between C-mAb Fab domains and FcRn. C) Interaction space of a generic Fab domain defined by combining all cross-links between the Fab domains of both C-mAb and H-mAb and FcRn. *Pale green*: α domains of FcRn, *light pink*: β2m, *wheat*: Fc-YTE.

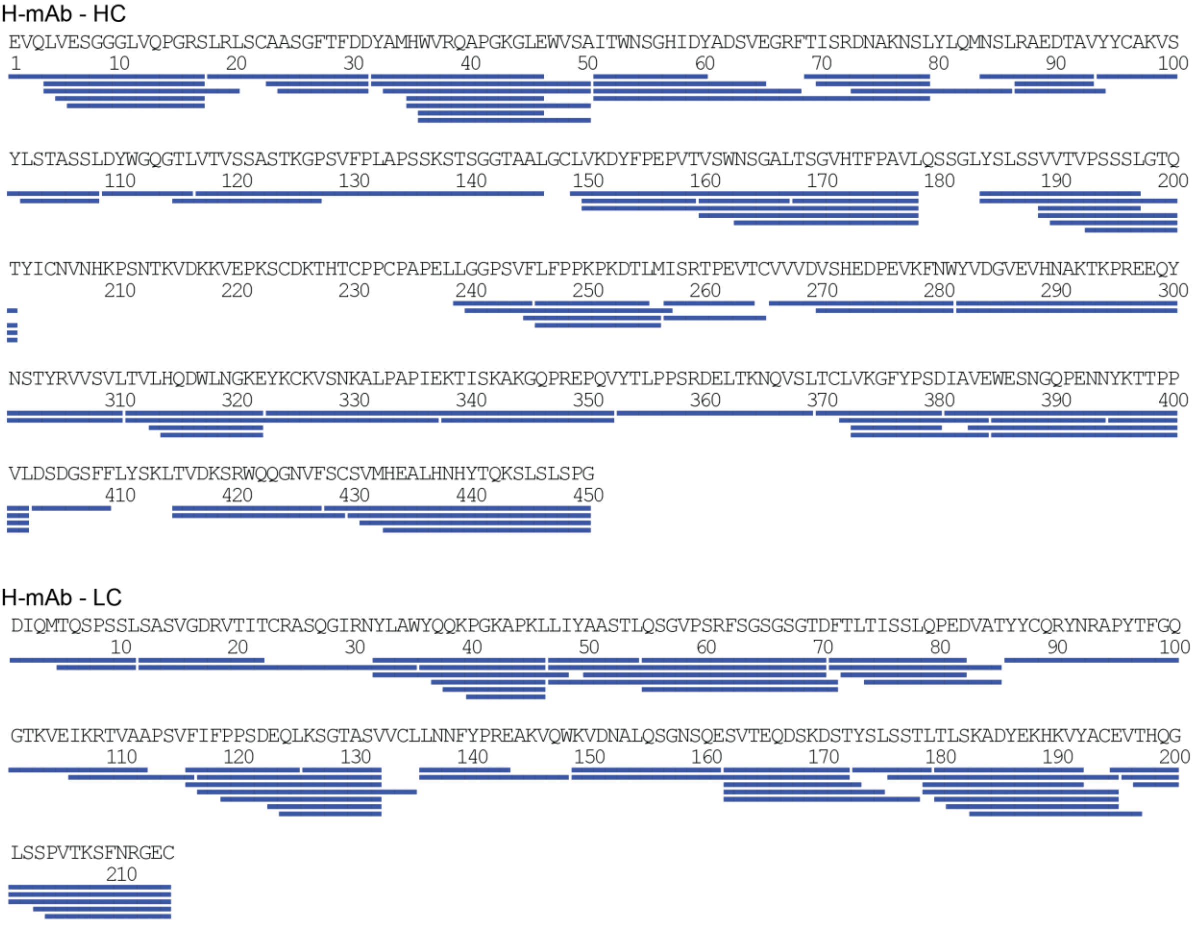

Supporting Figure S8: Effective HDX-MS coverage map of H-mAb. HDX data were obtained for 77 peptides covering 89.1% of the HC and for 51 peptides covering 100% of the LC.

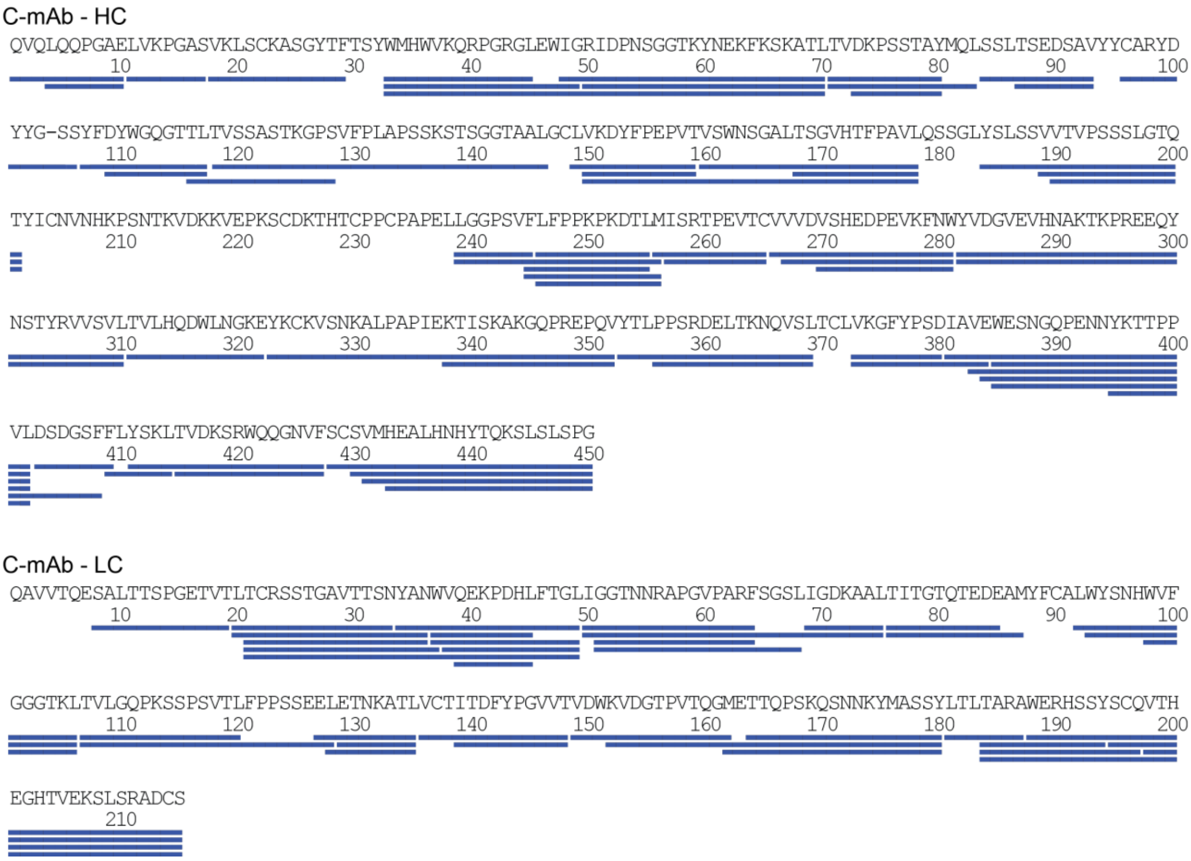

Supporting Figure S9: Effective HDX-MS coverage map of C-mAb. HDX data were obtained for 61 peptides covering 88.4% of the HC and for 39 peptides covering 94.9% of the LC.

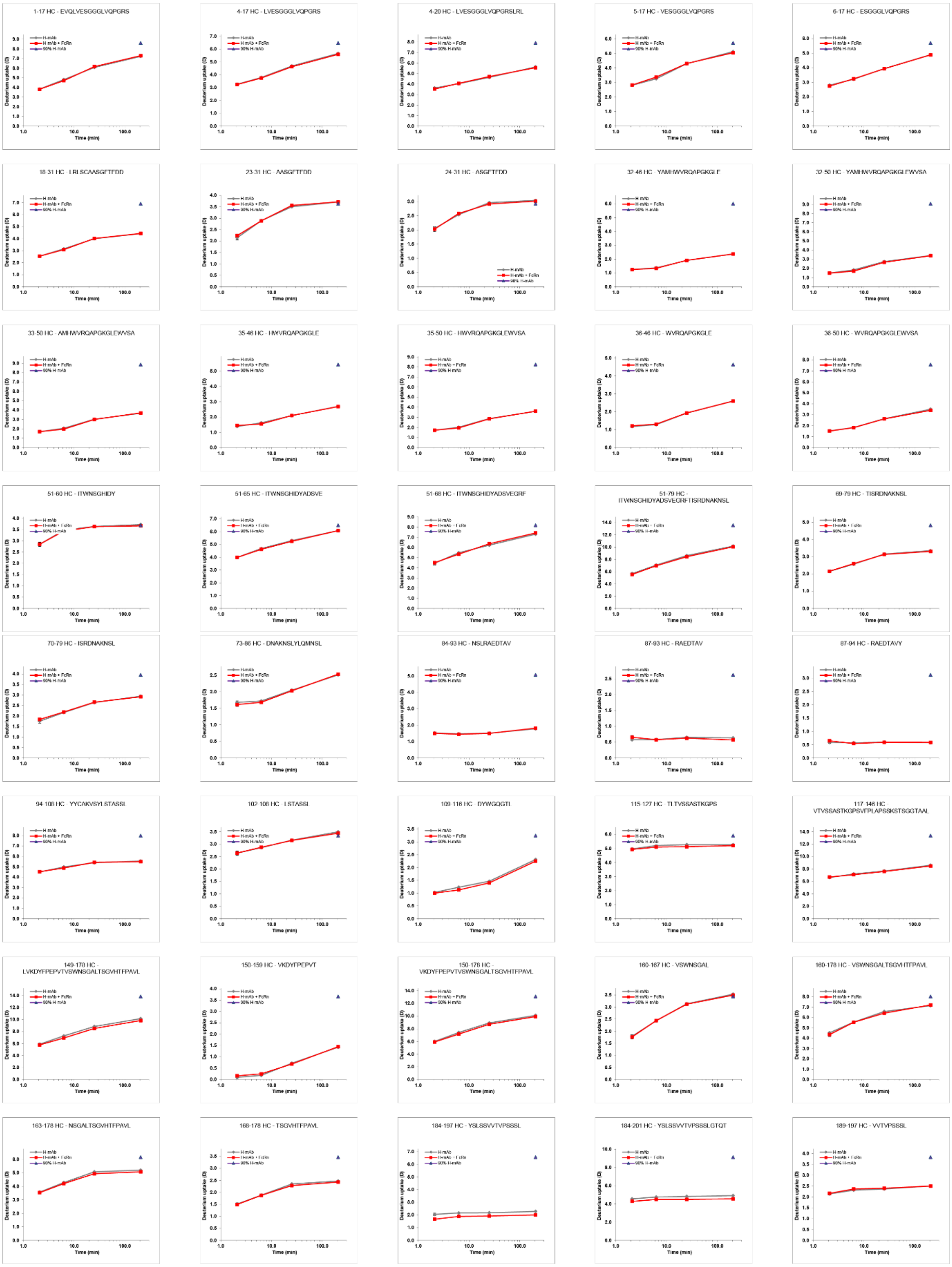
1-17 HC - EVQLVESGGGLVQPGRS
4-17 HC - LVESGGGLVQPGRS
4-20 HC - LVESGGGLVQPGRSLRL
5-17 HC - VESGGGLVQPGRS
6-17 HC - ESGGGLVQPGRS
18-31 HC - LRLSCAASGFTFDD
23-31 HC - AASGFTFDD
24-31 HC - ASGFTFDD
32-46 HC - YAMHWVRQAPGKGLE
32-50 HC - YAMHWVRQAPGKGLEWVSA
33-50 HC - AMHWVRQAPGKGLEWVSA
35-46 HC - HWVRQAPGKGLE
35-50 HC - HWVRQAPGKGLEWVSA
36-46 HC - WVRQAPGKGLE
36-50 HC - WVRQAPGKGLEWVSA
51-60 HC - ITWNSGHIDY
51-65 HC - ITWNSGHIDYADSVE
51-68 HC - ITWNSGHIDYADSVEGRF
51-79 HC - ITWNSGHIDYADSVEGRFTISRDNAKNSL
69-79 HC - TISRDNAKNSL
70-79 HC - ISRDNAKNSL
73-86 HC - DNAKNSLYLQMNSL
84-93 HC - NSLRAEDTAV
87-93 HC - RAEDTAV
87-94 HC - RAEDTAVY
94-108 HC - YYCAKVSYLSTASSL
102-108 HC - LSTASSL
109-116 HC - DYWGQGTL
115-127 HC - TLTVSSASTKGPS
117-146 HC - VTVSSASTKGPSVFPLAPSSKSTSGGTAAL
149-178 HC - LVKDYFPEPVTVSWNSGALTSGVHTFPAVL
150-159 HC - VKDYFPEPVT
150-178 HC - VKDYFPEPVTVSWNSGALTSGVHTFPAVL
160-167 HC - VSWNSGAL
160-178 HC - VSWNSGALTSGVHTFPAVL
163-178 HC - NSGALTSGVHTFPAVL
168-178 HC - TSGVHTFPAVL
184-197 HC - YSLSSVVTVPSSSL
184-201 HC - YSLSSVVTVPSSSLGTQT
189-197 HC - VVTVPSSSL
H mAb
H mAb + FcRn
90% H mAb
Deuterium uptake (D)
Time (min)

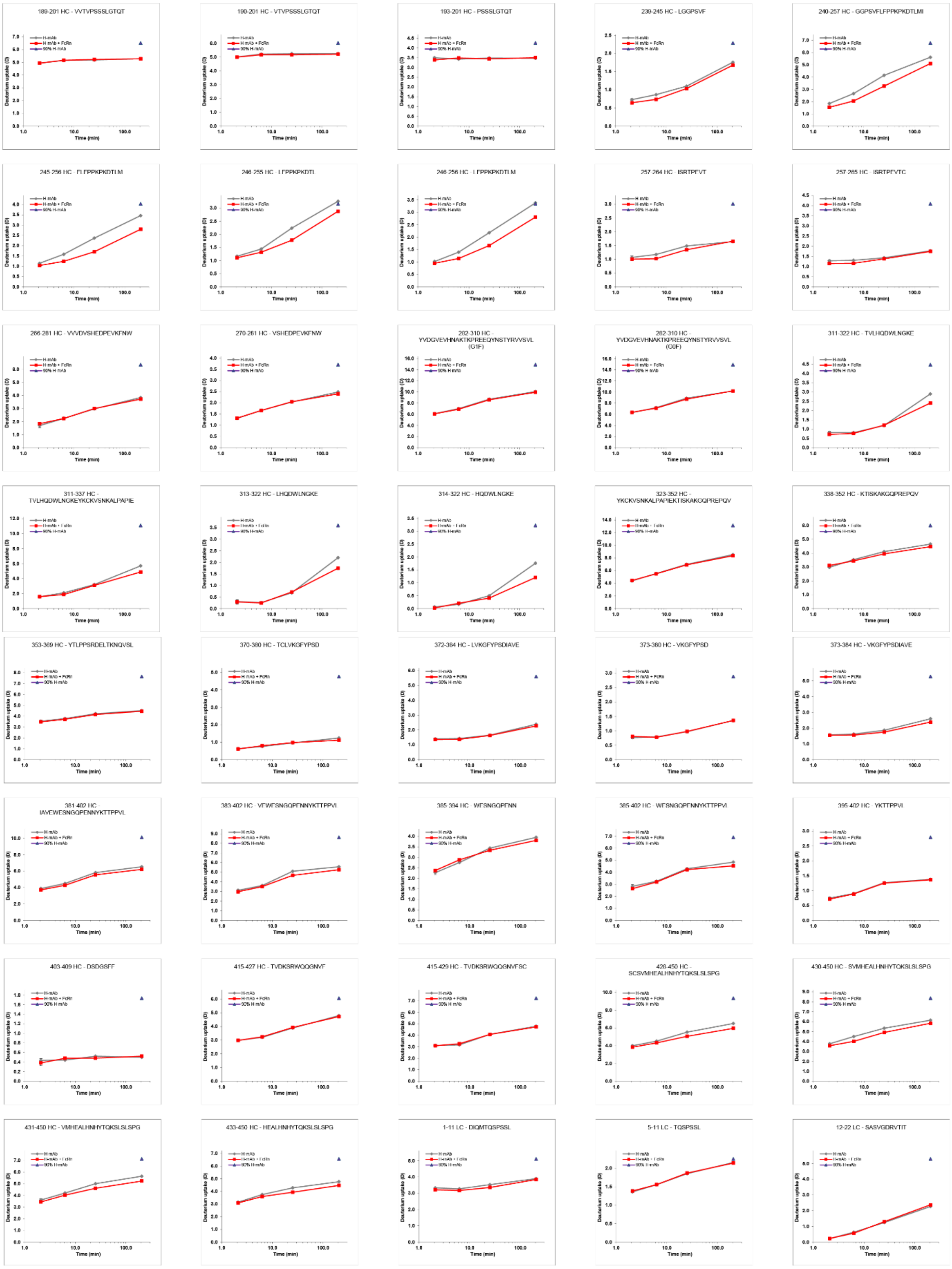
189-201 HC - VVTVPSSSLGTQT
190-201 HC - VTVPSSSLGTQT
193-201 HC - PSSSLGTQT
239-245 HC - LGGPSVF
240-257 HC - GGPSVFLFPPKPKDTLMI
245-256 HC - FLFPPKPKDTLM
246-255 HC - LFPPKPKDTL
246-256 HC - LFPPKPKDTLM
257-264 HC - ISRTPEVT
257-265 HC - ISRTPEVTC
266-281 HC - VVVDVSHEDPEVKFNW
270-281 HC - VSHEDPEVKFNW
282-310 HC - YVDGVEVHNAKTKPREEQYNSTYRVVSVL (G1F)
282-310 HC - YVDGVEVHNAKTKPREEQYNSTYRVVSVL (G0F)
311-322 HC - TVLHQDWLNGKE
311-337 HC - TVLHQDWLNGKEYKCKVSNKALPAPIE
313-322 HC - LHQDWLNGKE
314-322 HC - HQDWLNGKE
323-352 HC - YKCKVSNKALPAPIEKTISKAKGQPREPQV
338-352 HC - KTISKAKGQPREPQV
353-369 HC - YTLPPSRDELTKNQVSL
370-380 HC - TCLVKGFYPSD
372-384 HC - LVKGFYPSDIAVE
373-380 HC - VKGFYPSD
373-384 HC - VKGFYPSDIAVE
381-402 HC - IAVEWESNGQPENNYKTTPPVL
383-402 HC - VEWESNGQPENNYKTTPPVL
385-394 HC - WESNGQPENN
385-402 HC - WESNGQPENNYKTTPPVL
395-402 HC - YKTTPPVL
403-409 HC - DSDGSFF
415-427 HC - TVDKSRWQQGNVF
415-429 HC - TVDKSRWQQGNVFSC
426-450 HC - SCSVMHEALHNHYTQKSLSLSPG
430-450 HC - SVMHEALHNHYTQKSLSLSPG
431-450 HC - VMHEALHNHYTQKSLSLSPG
433-450 HC - HEALHNHYTQKSLSLSPG
1-11 LC - DIQMTQSPSSL
5-11 LC - TQSPSSL
12-22 LC - SASVGDRVTIT

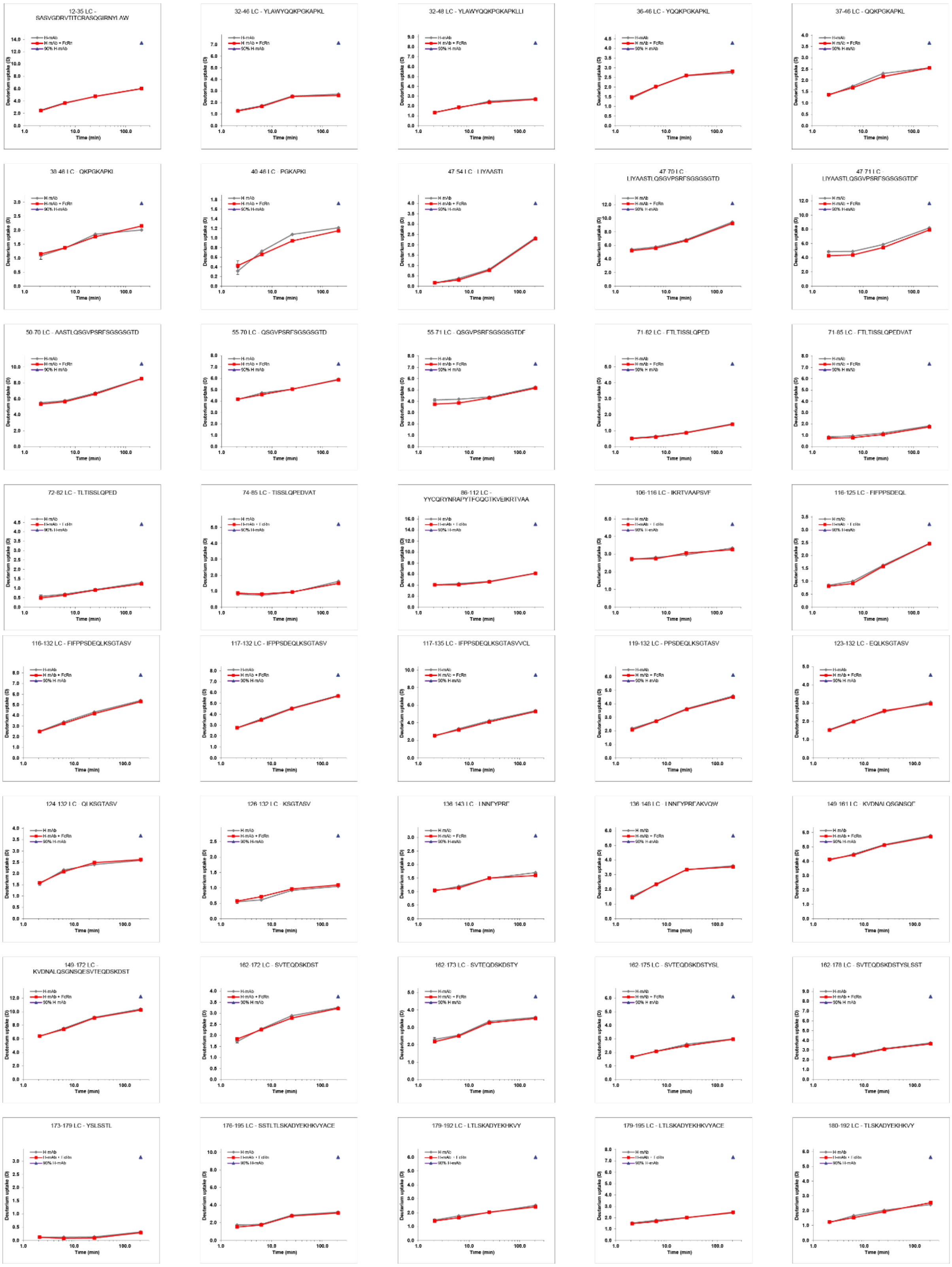
12-35 LC - SASVGDRVTITCRASQGIRNYLAW
32-46 LC - YLAWYQQKPGKAPKL
32-48 LC - YLAWYQQKPGKAPKLLI
36-46 LC - YQQKPGKAPKL
37-46 LC - QQKPGKAPKL
38-46 LC - QKPGKAPKL
40-46 LC - PGKAPKL
47-54 LC - LIYAASTL
47-70 LC - LIYAASTLQSGVPSRFSGSGSGTD
47-71 LC - LIYAASTLQSGVPSRFSGSGSGTDF
50-70 LC - AASTLQSGVPSRFSGSGSGTD
55-70 LC - QSGVPSRFSGSGSGTD
55-71 LC - QSGVPSRFSGSGSGTDF
71-82 LC - FTLTISSLQPED
71-85 LC - FTLTISSLQPEDVAT
72-82 LC - TLTISSLQPED
74-85 LC - TISSLQPEDVAT
86-112 LC - YYCQRYNRAPYTFGQGTKVEIKRTVAA
106-116 LC - IKRTVAAPSVF
116-125 LC - FIFPPSDEQL
116-132 LC - FIFPPSDEQLKSGTASV
117-132 LC - IFPPSDEQLKSGTASV
117-135 LC - IFPPSDEQLKSGTASVVCL
119-132 LC - PPSDEQLKSGTASV
123-132 LC - EQLKSGTASV
124-132 LC - QLKSGTASV
126-132 LC - KSGTASV
136-143 LC - LNNFYPRE
136-148 LC - LNNFYPREAKVQW
149-161 LC - KVDNALQSGNSQE
149-172 LC - KVDNALQSGNSQESVTEQDSKDST
162-172 LC - SVTEQDSKDST
162-173 LC - SVTEQDSKDSTY
162-175 LC - SVTEQDSKDSTYSL
162-178 LC - SVTEQDSKDSTYSLSST
173-179 LC - YSLSSTL
176-195 LC - SSTLTLSKADYEKHKVYACE
179-192 LC - LTLSKADYEKHKVY
179-195 LC - LTLSKADYEKHKVYACE
180-192 LC - TLSKADYEKHKVY
H-mAb
H-mAb + FcRn
90% H-mAb
Deuterium uptake (D)
Time (min)

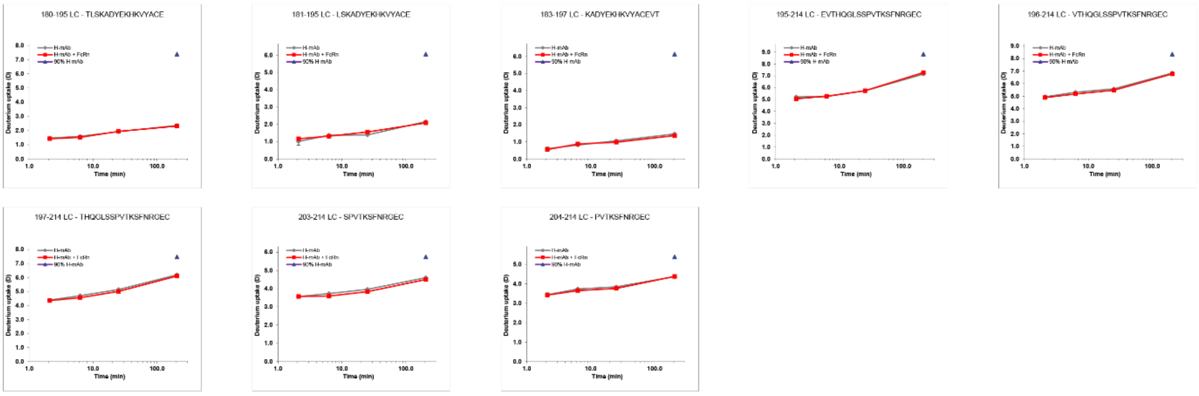

Supporting Figure S10: HDX plots of H-mAb in the absence and presence of FcRn. Absolute deuterium incorporation is plotted as a function of time for H-mAb (*grey* curves) and H-mAb in the presence of FcRn (*red* curves). Maximally labelled (90 %) control samples are plotted as *purple* triangles at the longest time points. SD is plotted as error bars (only slightly visible). (n = 1 for all data points, except the first time point, where n=3).

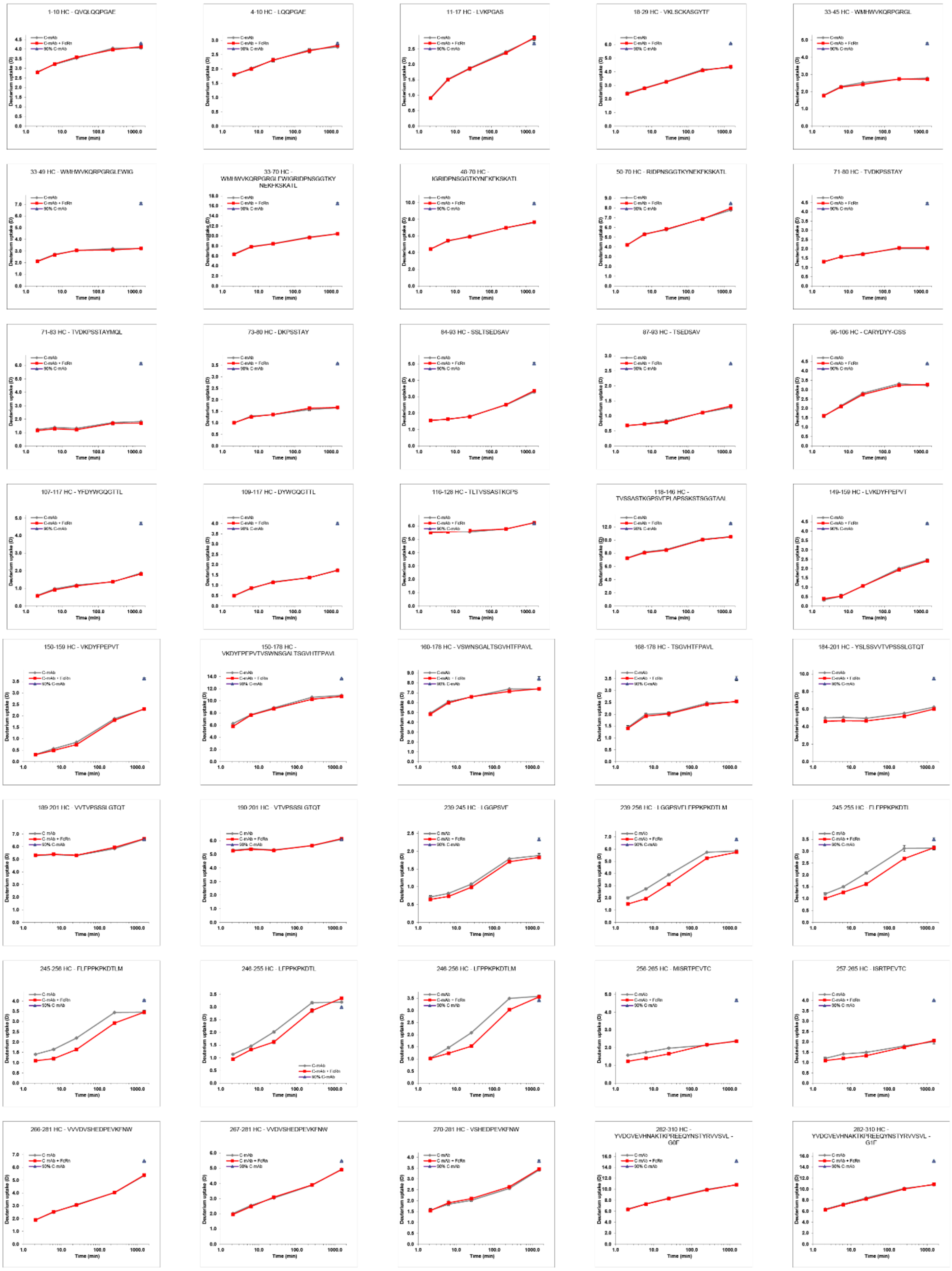
1-10 HC - QVQLQQPGAE
4-10 HC - LQQPGAE
11-17 HC - LVKPGAS
18-29 HC - VKLSCKASGYTF
33-45 HC - WMHWVKQRPGRGL
33-49 HC - WMHWVKQRPGRGLEWIG
33-70 HC - WMHWVKQRPGRGLEWIGRIDPNSGGTKYNEKFKSKATL
48-70 HC - IGRIDPNSGGTKYNEKFKSKATL
50-70 HC - RIDPNSGGTKYNEKFKSKATL
71-80 HC - TVDKPSSTAY
71-83 HC - TVDKPSSTAYMQL
73-80 HC - DKPSSTAY
84-93 HC - SSLTSEDSAV
87-93 HC - TSEDSAV
96-106 HC - CARYDYY-CSS
107-117 HC - YFDYWGQGTTL
109-117 HC - DYWGQGTTL
116-128 HC - TLTVSSASTKGPS
118-146 HC - TVSSASTKGPSVFPLAPSSKSTSGGTAAL
149-159 HC - LVKDYFPEPVT
150-159 HC - VKDYFPEPVT
150-178 HC - VKDYFPEPVTVSWNSGALTSGVHTFPAVL
160-178 HC - VSWNSGALTSGVHTFPAVL
168-178 HC - TSGVHTFPAVL
184-201 HC - YSLSSVVTVPSSSLGTQT
189-201 HC - VVTVPSSSLGTQT
190-201 HC - VTVPSSSLGTQT
239-245 HC - LGGPSVF
239-256 HC - LGGPSVFLFPPKPKDTLM
245-255 HC - FLFPPKPKDTL
245-256 HC - FLFPPKPKDTLM
246-255 HC - LFPPKPKDTL
246-256 HC - LFPPKPKDTLM
256-265 HC - MISRTPEVTC
257-265 HC - ISRTPEVTC
266-281 HC - VVVDVSHEDPEVKFNW
267-281 HC - VVDVSHEDPEVKFNW
270-281 HC - VSHEDPEVKFNW
282-310 HC - YVDGVEVHNAKTKPREEQYNSTYRVVSVL - G0F
282-310 HC - YVDGVEVHNAKTKPREEQYNSTYRVVSVL - G1F
C-mAb
C-mAb + FcRn
90% C-mAb
Deuterium uptake (D)
Time (min)

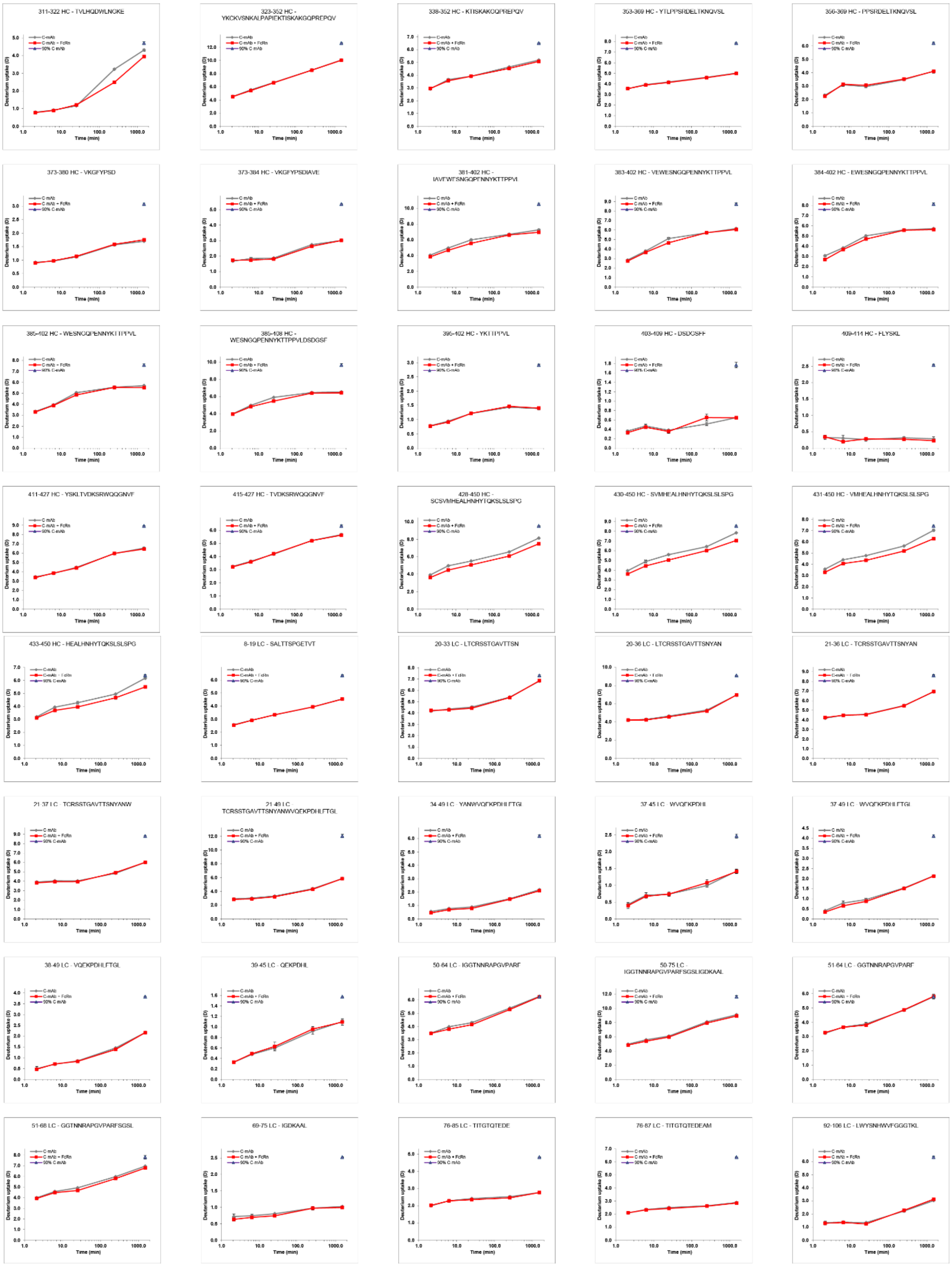
311-322 HC - TVLHQDWLNGKE
323-352 HC - YKCKVSNKALPAPIEKTISKAKGQPREPQV
338-352 HC - KTISKAKGQPREPQV
353-369 HC - YTLPPSRDELTKNQVSL
356-369 HC - PPSRDELTKNQVSL
373-380 HC - VKGFYPSD
373-384 HC - VKGFYPSDIAVE
381-402 HC - IAVEWESNGQPENNYKTTPPVL
383-402 HC - VEWESNGQPENNYKTTPPVL
384-402 HC - EWESNGQPENNYKTTPPVL
385-402 HC - WESNGQPENNYKTTPPVL
385-408 HC - WESNGQPENNYKTTPPVLDSDGSF
395-402 HC - YKTTPPVL
403-409 HC - DSDGSFF
409-414 HC - FLYSKL
411-427 HC - YSKLTVDKSRWQQGNVF
415-427 HC - TVDKSRWQQGNVF
428-450 HC - SCSVMHEALHNHYTQKSLSLSPG
430-450 HC - SVMHEALHNHYTQKSLSLSPG
431-450 HC - VMHEALHNHYTQKSLSLSPG
433-450 HC - HEALHNHYTQKSLSLSPG
8-19 LC - SALTTSPGETVT
20-33 LC - LTCRSSTGAVTTSN
20-36 LC - LTCRSSTGAVTTSNYAN
21-36 LC - TCRSSTGAVTTSNYAN
21-37 LC - TCRSSTGAVTTSNYANW
21-49 LC - TCRSSTGAVTTSNYANWVQEKPDHLFTGL
34-49 LC - YANWVQEKPDHLFTGL
37-45 LC - WVQEKPDHL
37-49 LC - WVQEKPDHLFTGL
38-49 LC - VQEKPDHLFTGL
39-45 LC - QEKPDHL
50-64 LC - IGGTNNRAPGVPARF
50-75 LC - IGGTNNRAPGVPARFSGSLIGDKAAL
51-64 LC - GGTNNRAPGVPARF
51-68 LC - GGTNNRAPGVPARFSGSL
69-75 LC - IGDKAAL
76-85 LC - TITGTQTEDE
76-87 LC - TITGTQTEDEAM
92-106 LC - LWYSNHWVFGGGTKL
C-mAb
C-mAb + FcRn
90% C-mAb
Deuterium uptake (D)
Time (min)

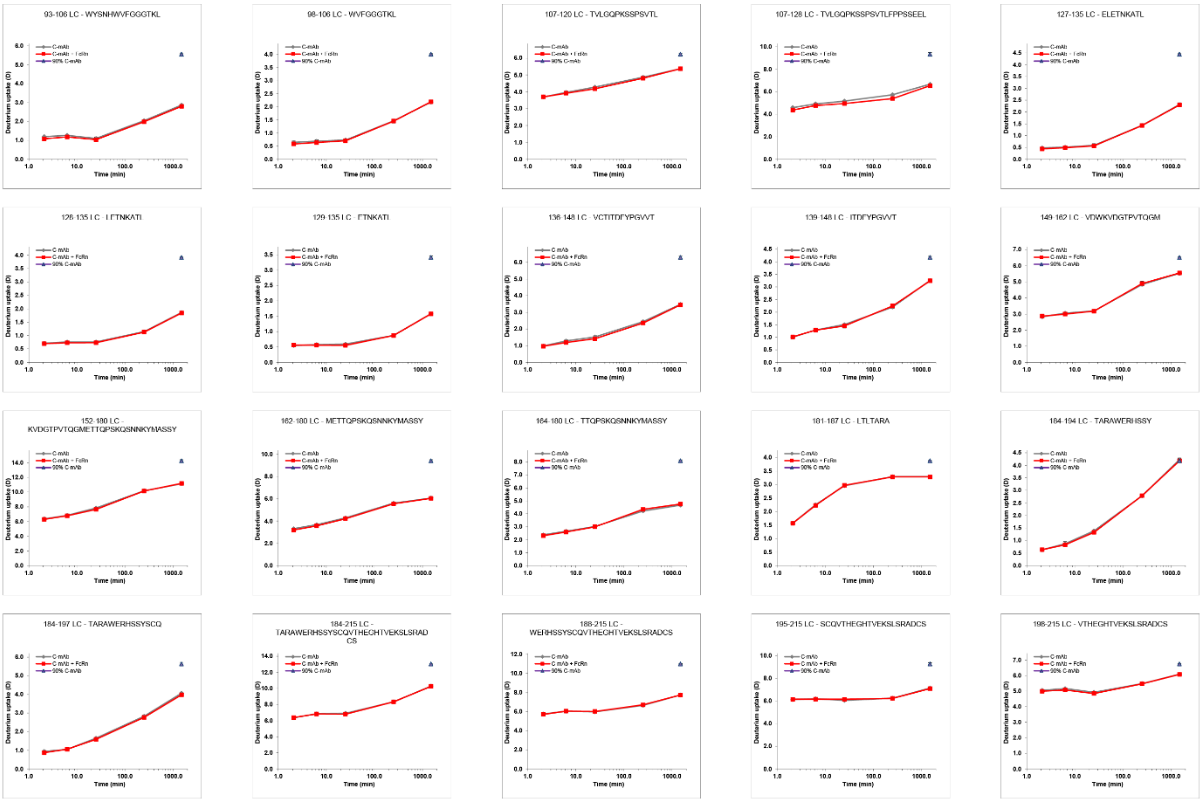

Supporting Figure S11 HDX plots of C-mAb in the absence and presence of FcRn. Absolute deuterium incorporation is plotted as a function of time for C-mAb (*grey* curves) and C-mAb in the presence of FcRn (*red* curves). Maximally labelled (90 %) control samples are plotted as *purple* triangles at the longest time points. SD is plotted as error bars (only slightly visible). (n=3).

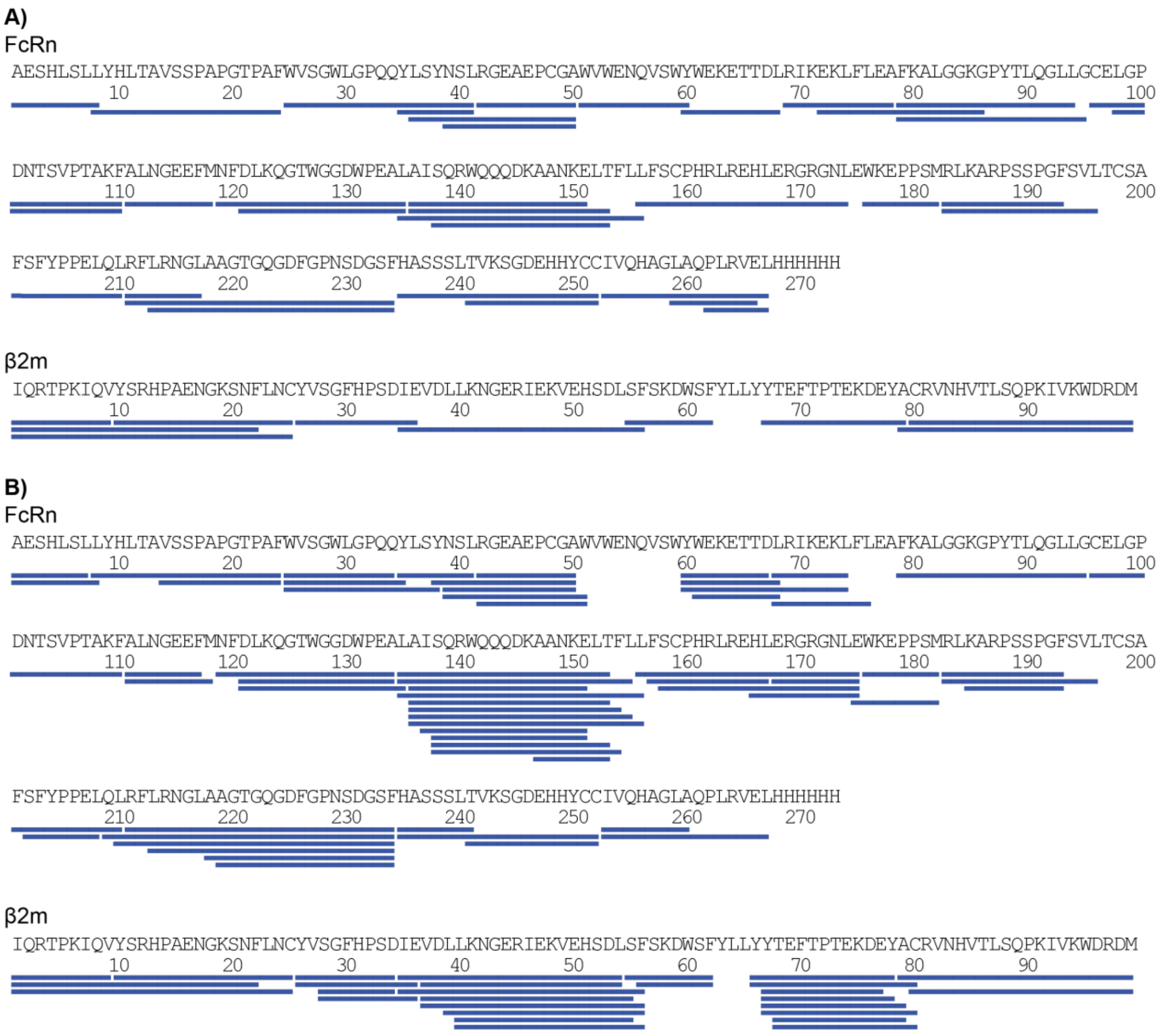

Supporting Figure S12: Effective HDX-MS coverage map of FcRn. Two separate data sets investigating two different exchange windows of FcRn in presence and absence of C-mAb and H-mAb were obtained. The first data set covered exchange times from 2.09 min to 1507.13 min (A), while the second data set covered exchange time from 13 s to 2.09 min (B). A) HDX data were obtained for 35 peptides covering 96.0% of the FcRn HC and for 10 peptides covering 96.0% of the β2m sequence. B) HDX data were obtained for 62 peptides covering 92.7% of the FcRn HC and for 28 peptides covering 97.0% of the β2m sequence.

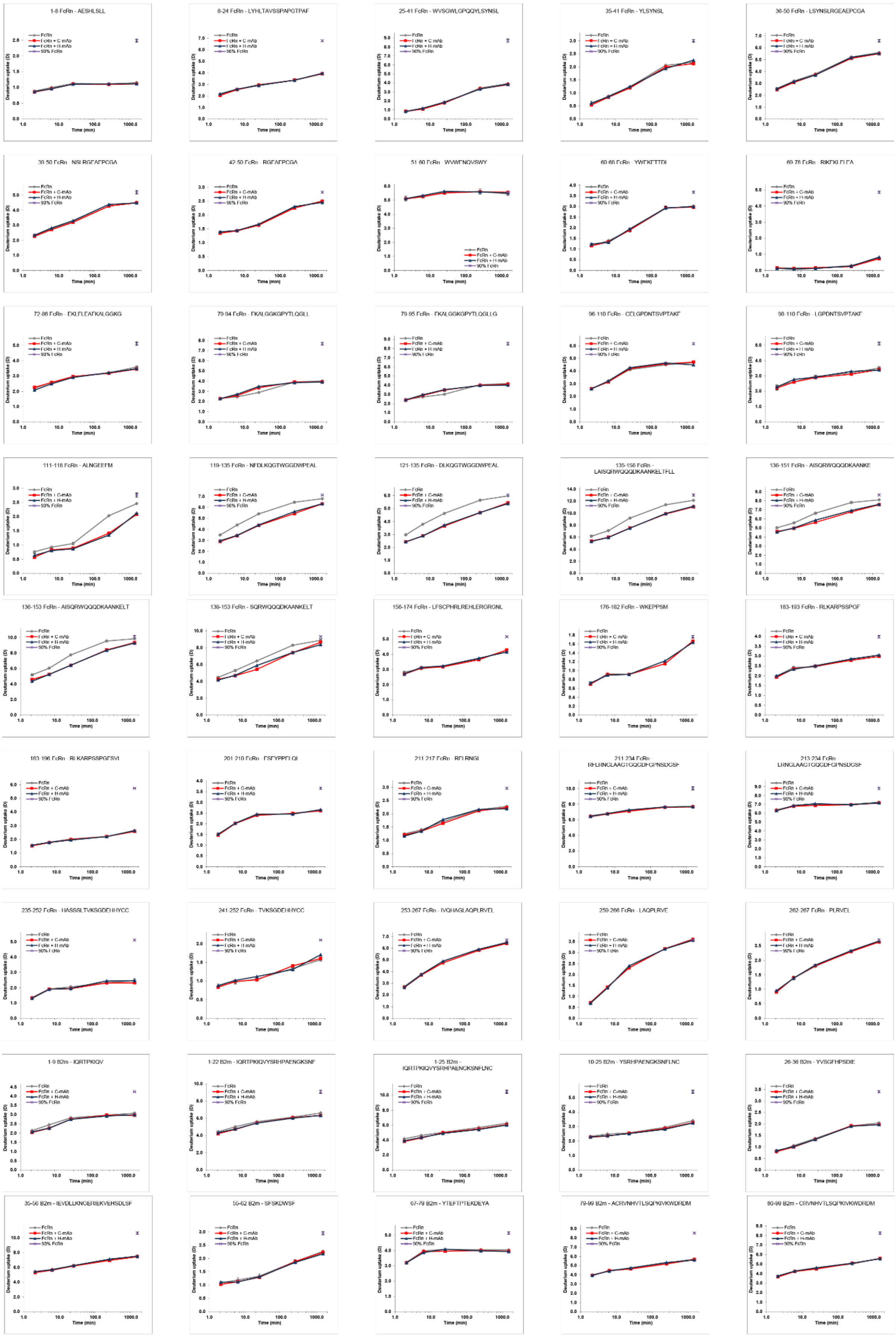
1-8 FcRn - AESHLSLL
8-24 FcRn - LYHLTAVSSPAPGTPAF
25-41 FcRn - WVSGWLGPQQYLSYNSL
35-41 FcRn - YLSYNSL
36-50 FcRn - LSYNSLRGEAEPCGA
39-50 FcRn - NSLRGEAEPCGA
42-50 FcRn - RGEAEPCGA
51-60 FcRn - WVWENQVSWY
60-66 FcRn - YWEKETTDL
69-78 FcRn - RIKEKLFLEA
72-86 FcRn - EKLFLEAFKALGGKG
79-94 FcRn - FKALGGKGPYTLQGLL
79-95 FcRn - FKALGGKGPYTLQGLLG
96-110 FcRn - CELGPDNTSVPTAKF
98-110 FcRn - LGPDNTSVPTAKF
111-118 FcRn - ALNGEEFM
119-135 FcRn - NFDLKQGTWGGDWPEAL
121-135 FcRn - DLKQGTWGGDWPEAL
135-156 FcRn - LAISQRWQQQDKAANKELTFLL
136-151 FcRn - AISQRWQQQDKAANKE
136-153 FcRn - AISQRWQQQDKAANKELT
138-153 FcRn - SQRWQQQDKAANKELT
156-174 FcRn - LFSCPHRLREHLERGRGNL
176-182 FcRn - WKEPPSM
183-193 FcRn - RLKARPSSPGF
183-196 FcRn - RLKARPSSPGFSVL
201-210 FcRn - FSFYPPELQL
211-217 FcRn - REERNGL
211-234 FcRn - REERNGLAAGTGQGDFGPNSDGSF
213-234 FcRn - ERNGLAAGTGQGDFGPNSDGSF
235-252 FcRn - HASSSLTVKSGDEHHYCC
241-252 FcRn - TVKSGDEHHYCC
253-267 FcRn - IVQHAGLAQPLRVEL
259-266 FcRn - LAQPLRVE
262-267 FcRn - PLRVEL
1-9 B2m - IQRTPKIQV
1-22 B2m - IQRTPKIQVYSRHPAENGKSNF
1-25 B2m - IQRTPKIQVYSRHPAENGKSNFLNC
10-25 B2m - YSRHPAENGKSNFLNC
26-36 B2m - YVSGFHPSDIE
35-56 B2m - IEVDLLKNGERIEKVEHSDLSF
55-62 B2m - SFSKDWSF
67-79 B2m - YTEFTPTEKDEYA
79-99 B2m - ACRVNHVTLSQPKIVKWDRDM
80-99 B2m - CRVNHVTLSQPKIVKWDRDM
FcRn
FcRn + C-mAb
FcRn + H-mAb
90% FcRn
Deuterium uptake (D)
Time (min)

Supporting Figure S13: HDX plots of FcRn in the absence and presence of either H-mAb or C-mAb (exchange time: 2.09 min to 1507.13 min). Absolute deuterium incorporation is plotted as a function of time for FcRn (*grey* curves), FcRn in the presence of C-mAb (*red lines*) and FcRn in the presence of H-mAb (*blue* curves). Maximally labelled (90 %) control samples are plotted as *purple* crosses at the longest time points. SD is plotted as error bars (only slightly visible). (n = 3 for all data points, except the last four time points of FcRn in presence of H-mAb, where n=1).

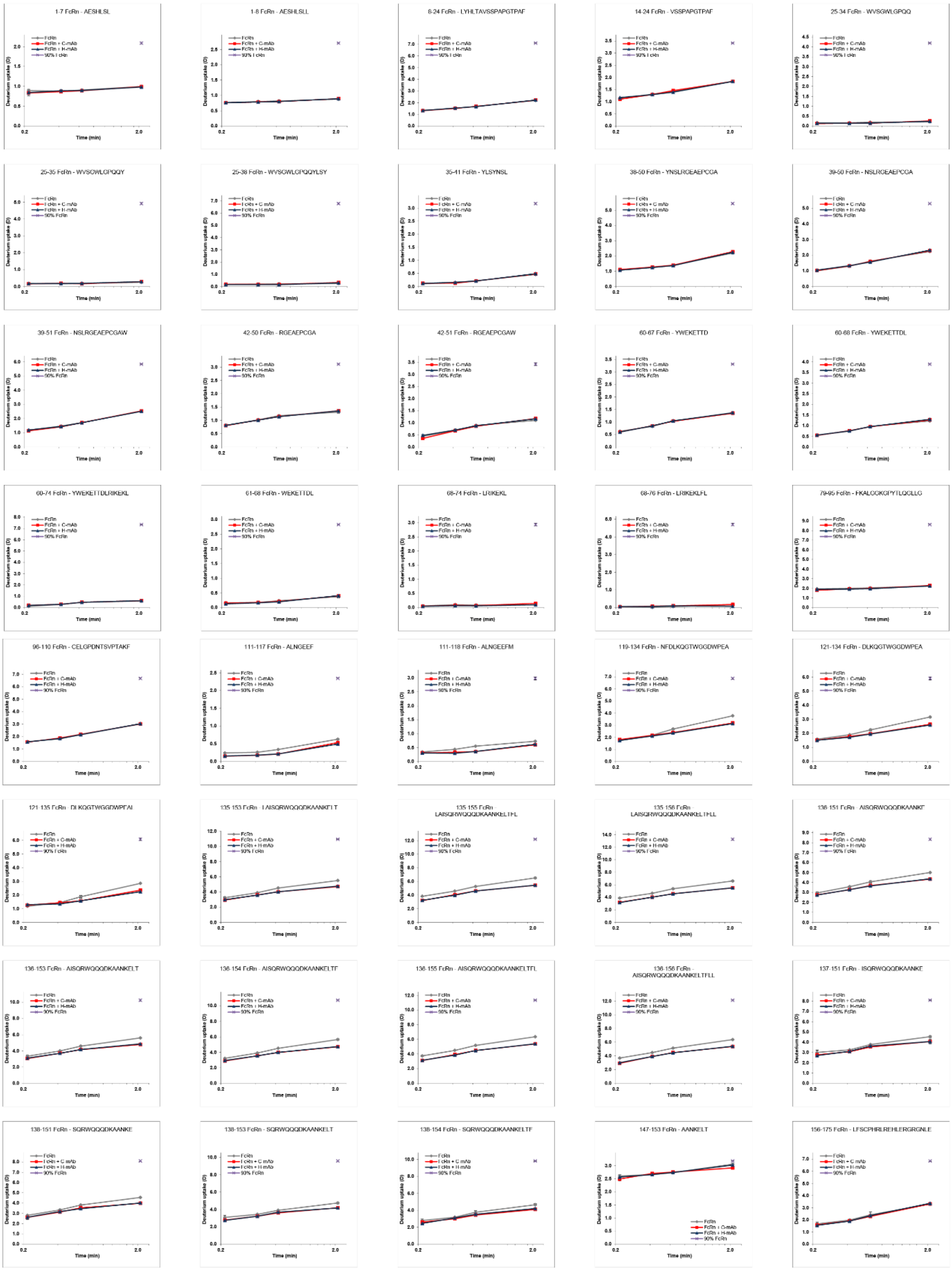
1-7 FcRn - AESHLSL
1-8 FcRn - AESHLSLL
6-24 FcRn - LYHLTAVSSPAPGTPAF
14-24 FcRn - VSSPAPGTPAF
25-34 FcRn - WVSGWLGPQQ
25-35 FcRn - WVSGWLGPQQY
25-38 FcRn - WVSGWLGPQQYLSY
35-41 FcRn - YLSYNSL
38-50 FcRn - YNSLRGEAEPCGA
39-50 FcRn - NSLRGEAEPCGA
39-51 FcRn - NSLRGEAEPCGAW
42-50 FcRn - RGEAEPCGA
42-51 FcRn - RGEAEPCGAW
60-67 FcRn - YWEKETTD
60-68 FcRn - YWEKETTDL
60-74 FcRn - YWEKETTDLRIKEKL
61-68 FcRn - WEKETTDL
68-74 FcRn - LRIKEKL
68-76 FcRn - LRIKEKLFL
79-95 FcRn - FKALGGKGPYTLQGLLG
96-110 FcRn - CELGPDNTSVPTAKF
111-117 FcRn - ALNGEEF
111-118 FcRn - ALNGEEFM
119-134 FcRn - NFDLKQGTWGGDWPEA
121-134 FcRn - DLKQGTWGGDWPEA
121-135 FcRn - DLKQGTWGGDWPEAL
135-153 FcRn - LAISQRWQQQDKAANKELT
135-155 FcRn LAISQRWQQQDKAANKELTFL
135-156 FcRn LAISQRWQQQDKAANKELTFLL
136-151 FcRn - AISQRWQQQDKAANKE
136-153 FcRn - AISQRWQQQDKAANKELT
136-154 FcRn - AISQRWQQQDKAANKELTF
136-155 FcRn - AISQRWQQQDKAANKELTFL
136-156 FcRn AISQRWQQQDKAANKELTFLL
137-151 FcRn - ISQRWQQQDKAANKE
138-151 FcRn - SQRWQQQDKAANKE
138-153 FcRn - SQRWQQQDKAANKELT
138-154 FcRn - SQRWQQQDKAANKELTF
147-153 FcRn - AANKELT
156-175 FcRn - LFSCPHRLREHLERGRGNLE
FcRn
FcRn + C-mAb
FcRn + H-mAb
90% FcRn
Deuterium uptake (D)
Time (min)

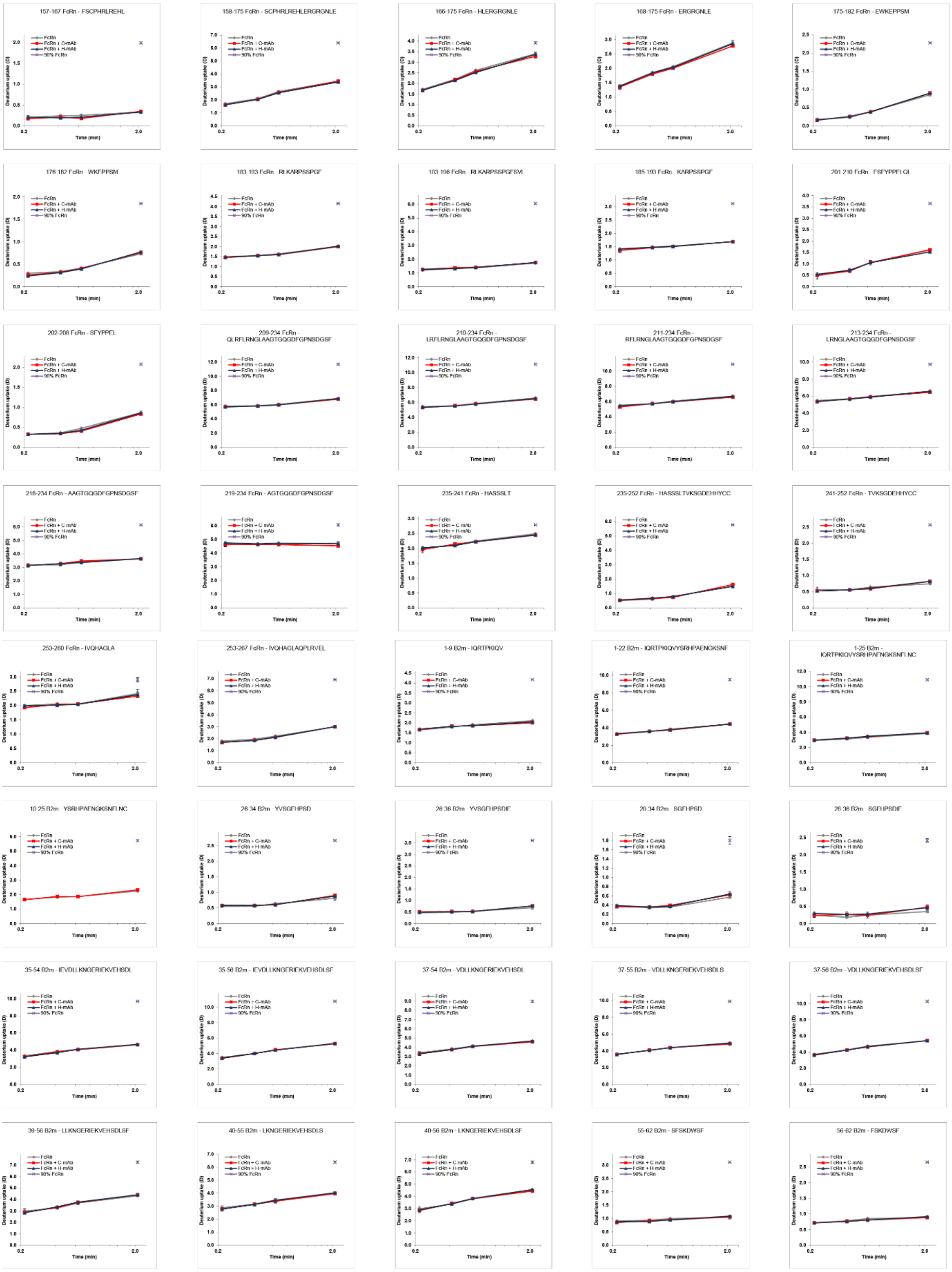
157-167 FcRn - FSCPHRLREHL
158-175 FcRn - SCPHRLREHLERGRGNLE
166-175 FcRn - HLERGRGNLE
168-175 FcRn - ERGRGNLE
175-182 FcRn - EWKEPPSM
176-182 FcRn - WKEPPSM
183-193 FcRn - RLKARPSSPGF
183-196 FcRn - RLKARPSSPGFSVL
185-193 FcRn - KARPSSPGF
201-210 FcRn - FSFYPPELQL
202-208 FcRn - SFYPPEL
209-234 FcRn - QLRFLRNGLAAGTGQGDFGPNSDGSF
210-234 FcRn - LRFLRNGLAAGTGQGDFGPNSDGSF
211-234 FcRn - RFLRNGLAAGTGQGDFGPNSDGSF
213-234 FcRn - LRNGLAAGTGQGDFGPNSDGSF
218-234 FcRn - AAGTGQGDFGPNSDGSF
219-234 FcRn - AGTGQGDFGPNSDGSF
235-241 FcRn - HASSSLT
235-252 FcRn - HASSSLTVKSGDEHHYCC
241-252 FcRn - TVKSGDEHHYCC
253-260 FcRn - IVQHAGLA
253-267 FcRn - IVQHAGLAQPLRVEL
1-9 B2m - IQRTPKIQV
1-22 B2m - IQRTPKIQVYSRHPAENGKSNF
1-25 B2m - IQRTPKIQVYSRHPAENGKSNFLNC
10-25 B2m - YSRHPAENGKSNFLNC
26-34 B2m - YVSGFHPSD
26-36 B2m - YVSGFHPSDIE
28-34 B2m - SGFHPSD
28-36 B2m - SGFHPSDIE
35-54 B2m - IEVDLLKNGERIEKVEHSDL
35-56 B2m - IEVDLLKNGERIEKVEHSDLSF
37-54 B2m - VDLLKNGERIEKVEHSDL
37-55 B2m - VDLLKNGERIEKVEHSDLS
37-56 B2m - VDLLKNGERIEKVEHSDLSF
39-56 B2m - LLKNGERIEKVEHSDLSF
40-55 B2m - LKNGERIEKVEHSDLS
40-56 B2m - LKNGERIEKVEHSDLSF
55-62 B2m - SFSKDWSF
56-62 B2m - FSKDWSF
FcRn
FcRn + C-mAb
FcRn + H-mAb
90% FcRn
Deuterium uptake (D)
Time (min)

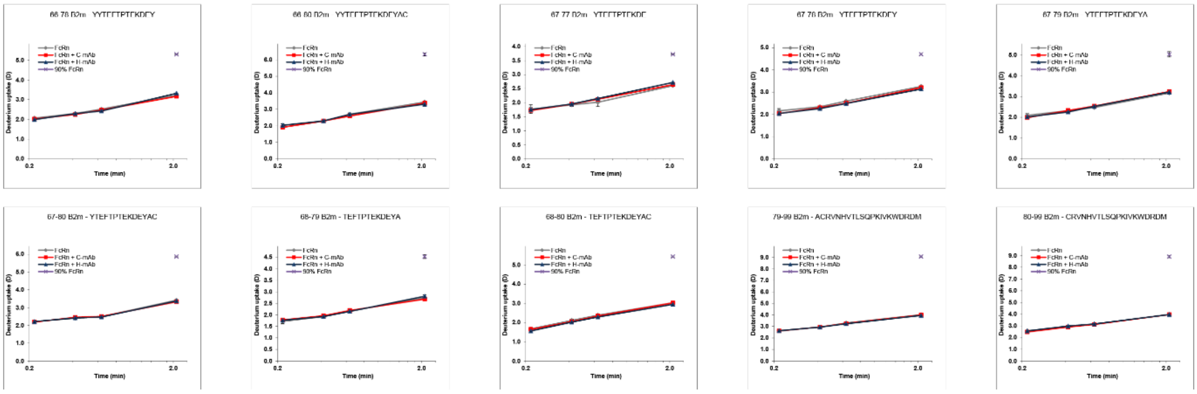

Supporting Figure S14: HDX plots of FcRn in the absence and presence of either H-mAb or C-mAb (exchange time: 13 s to 2.09 min). Absolute deuterium incorporation is plotted as a function of time for FcRn (*grey lines*), FcRn in the presence of C-mAb (*red lines*) and FcRn in the presence of H-mAb (*blue lines*). Maximally labelled (90 %) control samples are plotted as *purple crosses* at the longest time points. SD is plotted as error bars (only slightly visible). (n = 3 for all data points).

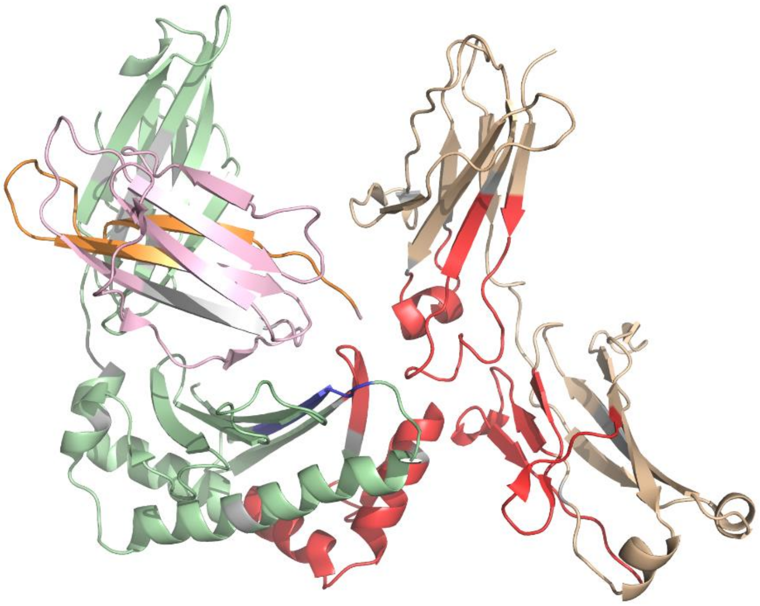

Supporting Figure S15: Conformational response of complex formation between FcRn and C-mAb. Differences in HDX mapped onto the crystal structure of FcRn in complex with Fc-YTE (α-domain: *pale green*, β2m: *light pink*, Fc-YTE: *wheat*, PDB ID: 4N0U). Regions in the α-domain and β2m of FcRn displaying a significant protection from exchange in the presence of H-mAb or C-mAb are colored *orange* and *red*, respectively. Regions displaying a significant deprotection from exchange in the presence of H-mAb or C-mAb are colored *blue.* Regions in the Fc-part of C-mAb displaying a significant protection from exchange in the presence of FcRn are colored *red*, *while* regions for which no HDX information could be obtained are colored *grey*.

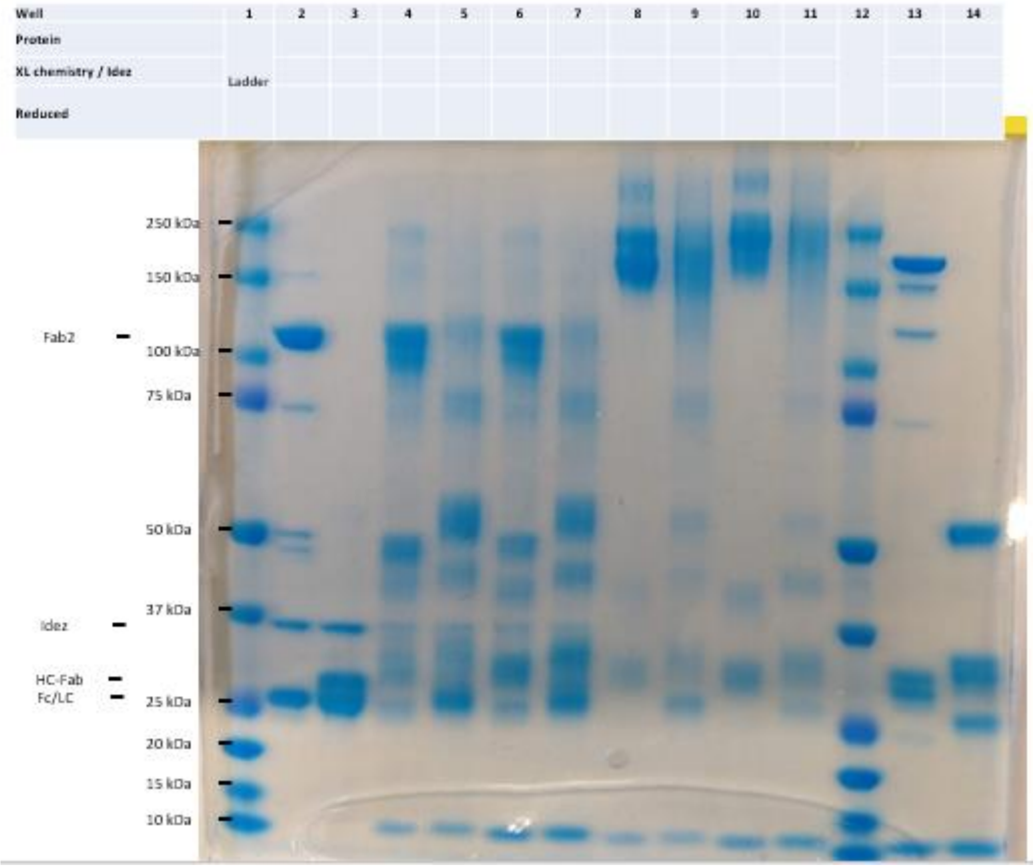

Supporting Figure S16: SDS-PAGE of IdeZ treated C-mAb. Well 2 (non-reduced conditions) and 3 (reduced conditions) illustrate the successful cleavage of C-mAb by IdeZ.

Supplemental References

1. Rice, P., Longden, I. & Bleasby, A. EMBOSS: the European Molecular Biology Open Software Suite. *Trends Genet* **16**, 276–7 (2000).

2. Oganesyan, V. *et al.* Structural insights into neonatal Fc receptor-based recycling mechanisms. *J Biol Chem* **289**, 7812–7824 (2014).

3. Altschul, S. F. *et al.* Gapped BLAST and PSI-BLAST: a new generation of protein database search programs. *Nucleic Acids Res* **25**, 3389–402 (1997).

4. Altschul, S. F. *et al.* Protein database searches using compositionally adjusted substitution matrices. *FEBS J* **272**, 5101–9 (2005).
